# Cu-Based Materials for Enhanced C_2+_ Product Selectivity in Photo-/Electro-Catalytic CO_2_ Reduction: Challenges and Prospects

**DOI:** 10.1007/s40820-023-01276-2

**Published:** 2024-01-04

**Authors:** Baker Rhimi, Min Zhou, Zaoxue Yan, Xiaoyan Cai, Zhifeng Jiang

**Affiliations:** 1 Institute for Energy Research, School of Chemistry and Chemical Engineering, Jiangsu University, Zhenjiang, 212013 People’s Republic of China; 2 School of Materials Science and Physics, China University of Mining and Technology, Xuzhou, 221116 People’s Republic of China

**Keywords:** Photocatalytic CO_2_ reduction, Cu-based materials, Electrocatalytic CO_2_ reduction

## Abstract

Carbon dioxide conversion into valuable products using photocatalysis and electrocatalysis is an effective approach to mitigate global environmental issues and the energy shortages. Among the materials utilized for catalytic reduction of CO_2_, Cu-based materials are highly advantageous owing to their widespread availability, cost-effectiveness, and environmental sustainability. Furthermore, Cu-based materials demonstrate interesting abilities in the adsorption and activation of carbon dioxide, allowing the formation of C_2+_ compounds through C–C coupling process. Herein, the basic principles of photocatalytic CO_2_ reduction reactions (PCO_2_RR) and electrocatalytic CO_2_ reduction reaction (ECO_2_RR) and the pathways for the generation C_2+_ products are introduced. This review categorizes Cu-based materials into different groups including Cu metal, Cu oxides, Cu alloys, and Cu SACs, Cu heterojunctions based on their catalytic applications. The relationship between the Cu surfaces and their efficiency in both PCO_2_RR and ECO_2_RR is emphasized. Through a review of recent studies on PCO_2_RR and ECO_2_RR using Cu-based catalysts, the focus is on understanding the underlying reasons for the enhanced selectivity toward C_2+_ products. Finally, the opportunities and challenges associated with Cu-based materials in the CO_2_ catalytic reduction applications are presented, along with research directions that can guide for the design of highly active and selective Cu-based materials for CO_2_ reduction processes in the future.

## Highlights

- The latest advancements in Cu-based catalysts for photocatalytic and electrocatalytic CO_2_ reduction into C_2+_ products are reported.
- The relationship between the Cu surfaces and their efficiency in photocatalytic and electrocatalytic CO_2_ reduction is emphasized.
- The opportunities and challenges associated with Cu-based materials in the CO_2_ catalytic reduction applications are presented.

## Graphical abstract

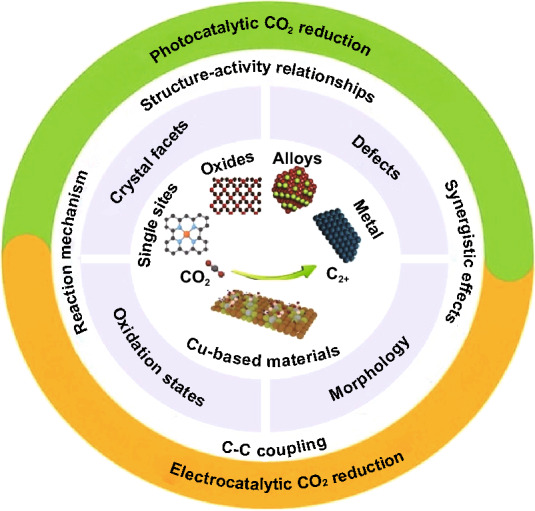

## Introduction

Over the past few years, there has been a significant increase in the global energy consumption rate, resulting in massive use of fossil fuels such as natural gas, oil, and coal, which presently account for approximately 85% of primary energy supply [1–3]. Since fossil fuels, a non-renewable resource, are finite, their overconsumption has caused energy shortage issues. Moreover, the burning of fossil fuels is connected with excessive emissions of hazardous gases, including NO_*x*_, SO_2_, and CO_2_. CO_2_ is one of the primary gases that contribute to greenhouse effect, which results in rising global temperatures, ocean acidification, climatic variation, and so on [4–6]. Therefore, the mitigation of CO_2_ emissions is critical in tackling the issues of global warming and energy depletion. In this context, numerous research studies have focused on developing effective ways for carrying out the artificial conversion of CO_2_ in order to address these issues. It is intended to utilize CO_2_ as a carbon feedstock in order to produce more value and useful hydrocarbon products through catalytic reactions. These products may then be used in a variety of industrial processes. These initiatives are crucial for promoting a sustainable approach toward energy production while also lowering the harmful impacts of carbon emissions on the environment.

As a chemical feedstock, CO_2_ has attracted the attention of researchers across several fields, including thermocatalytic, electrocatalytic, and photocatalytic CO_2_ conversion. However, the high cost of thermochemical carbon dioxide conversion limits its practical use. As a consequence, researchers are investigating alternative approaches for catalytically converting CO_2_ into valuable products while reducing the cost of CO_2_ conversion, such as wind and solar energy. Sustainable CO_2_ conversion methods that use renewable energy sources at room temperature, such as photocatalytic CO_2_ reduction reactions (PCO_2_RR) and electrocatalytic CO_2_ reduction reactions (ECO_2_RR), have the potential to be more practical and cost-effective alternatives for CO_2_ conversion [7, 8]. These methods differ in the source of electrons involved in the catalysis process, and the mechanisms by which this conversion is achieved are distinct: Photons are the primary source of electrons in photocatalysis, while an external electric field drives electrons in electrocatalysis.

Although the methodologies and underlying principles of PCO_2_RR and ECO_2_RR processes are different, their nature is essentially identical. These approaches employ catalysts to transform CO_2_ into high value-added products; nevertheless, significant challenges remain in terms of CO_2_ conversion efficiency and the selectivity of resulted products due to the inert nature of CO_2_ molecules and the intricate nature of the process. This outcome arises from the thermodynamic stability and chemical inertness of CO_2_, which is a linear molecule with completely oxidized carbon and an average C=O double bond energy of up to 804.4 kJ mol^−1^ (at 298 K) that requires substantial energy to break its carbon–oxygen (C–O) bonds [9, 10]. The thermodynamically stable nature of CO_2_ makes it challenging to catalyze the CO_2_ conversion process, and the inertness of its molecular structure limits the number of catalytic sites and affects the reaction selectivity, leading to low yields of value-added products. Additionally, the complexity of the CO_2_ conversion process makes it difficult to optimize conditions for high activity and product selectivity, which limits the overall CO_2_ conversion efficiency. These challenges underline the need for improvements in CO_2_ conversion technologies, particularly in respect to developing efficient catalysts that enhance CO_2_ adsorption and activation to promote higher activity and selectivity of the process. Meanwhile, finding the right catalyst for each method requires a deep understanding of the underlying mechanism of each approach.

**Fig. 1:**
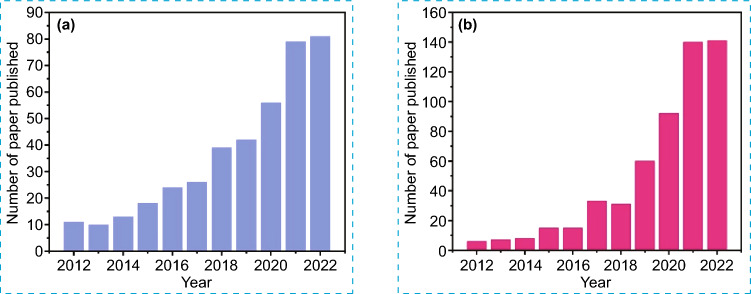
The number of published articles between 2012 and 2022 retrieved from the Web of Science database: **a** photocatalytic CO_2_ reduction to C_2+_ products and **b** electrocatalytic CO_2_ reduction to C_2+_ products

In general, the CO_2_ reduction reaction (CO_2_RR) is a complex chemical process that consists of a series of electron transfer steps, hydrogenation, C–C bond coupling, and intermediate compounds. At present, products obtained from CO_2_RR are often classified into C_1_ and multi-carbon C_2+_ compounds. The representative C_1_ compounds such as methane (CH_4_), carbon monoxide (CO), methanol (CH_3_OH), formaldehyde (CH_2_O), and formic acid (HCOOH) have been extensively researched, whereas the formation of C_2+_ products, including but not limited to ethylene (C_2_H_4_), ethane (C_2_H_6_), ethanol (C_2_H_5_OH), and propanol (C_3_H_7_OH), poses a significant challenge because of the complex reaction pathways along with competitive reactions. Table 1 summarizes the possible reactions of CO_2_RR to C_1_ and C_2+_ products and their market prices [11–14]. Based on the market price point of view, C_2+_ products are more attractive in comparison with C_1_ products. Thus, it is effective to produce C_2+_ through a single process via the CO_2_RR. Figure 1 displays the annual count of scholarly research articles and review papers published within the timeframe of 2012–2022. The data collected from the Web of Science database indicates an increasing interest among the scholarly community in the topic of photocatalytic and electrocatalytic CO_2_ conversion into highly valuable C_2+_ compounds. This highlights the significance of research in this area as it could potentially offer solutions for combating climate change and energy shortage.

**Table 1:** Products obtained from CO_2_ reduction with their redox potentials relative to NHE (at pH 7) and their market prices

Reaction	E^0^_redo*x*_ vs. NHE (V)	Product	Market price ($/kg)	Equations
CO_2_ + e^−^ $$\to$$ CO_2_^· -^	− 1.90	CO_2_^· -^	–	(1)
CO_2_ + 2H^+^ + 2e^−^ $$\to$$ CO_(g)_ + H_2_O	− 0.53	Carbon monoxide	0.06	(2)
CO_2_ + 2H^+^ + 2e^−^ $$\to$$ HCOOH_(aq)_	− 0.61	Formic acid	0.74	(3)
CO_2_ + 4H^+^ + 4e^−^ $$\to$$ HCHO_(aq)_ + H_2_O	− 0.48	Formaldehyde	0.8	(4)
CO_2_ + 6H^+^ + 6e^−^ $$\to$$ CH_3_OH_(aq)_ + H_2_O	− 0.38	Methanol	0.58	(5)
CO_2_ + 8H^+^ + 8e^−^ $$\to$$ CH_4(g)_ + 2H_2_O	− 0.24	Methane	0.18	(6)
2CO_2_ + 8H^+^ + 8e^−^ $$\to$$ CH_3_COOH_(aq)_ + 2H_2_O	− 0.31	Acetic acid	2.9	(7)
2CO_2_ + 12H^+^ + 12e^−^ $$\to$$ C_2_H_4(g)_ + 4H_2_O	− 0.34	Ethylene	1.3	(8)
2CO_2_ + 12H^+^ + 12e^−^ $$\to$$ C_2_H_5_OH_(aq)_ + 3H_2_O	− 0.32	Ethanol	1.0	(9)
2CO_2_ + 14H^+^ + 14e^−^ $$\to$$ C_2_H_6(g)_ + 4H_2_O	− 0.51	Ethane	4.0	(10)
3CO_2_ + 18H^+^ + 18e^−^ $$\to$$ C_3_H_7_OH_(aq)_ + 5H_2_O	− 0.32	Propanol	1.43	(11)
2H^+^ + 2e^−^ $$\to$$ H_2(g)_	− 0.41	Hydrogen	–	(12)

Over the recent decades, significant efforts have been made to enhance the selectivity of C_2+_ products and investigate the various factors that influence their distribution. Copper (Cu) displayed distinctive properties in converting CO_2_ into C_2+_ chemicals such as ethylene, ethane, and ethanol, in both photocatalytic and electrocatalytic CO_2_ reduction [15–19]. Copper is a readily available and cost-effective element that is abundant in the earth’s reserves. It possesses multiple oxidation states, which allow for the formation of various copper-based materials such as cuprous oxide (Cu_2_O) and copper oxide (CuO). CuO and Cu_2_O semiconductors possess narrow bandgaps of 1.7 and 2.2 eV, respectively, making them efficient in capturing visible light energy [20, 21]. Additionally, the sufficiently negative conduction band (CB) position and the ability to effectively adsorb CO_2_ make these materials efficient photoactive catalysts for CO_2_ reduction [22, 23]. For instance, several structural factors, including surface states, surface defects, particle size, morphology, and crystal facet have been identified to affect the catalytic performance of Cu-based materials in PCO_2_RR and ECO_2_RR [24–27]. However, the synergy between these factors further complicates the understanding control of CO_2_RR product selectivity. According to a recent report, the valance state of the Cu^*n*+ ^ (0 < *n* < 2) that is present on the surface of catalysts is a crucial factor in governing C–C coupling in PCO_2_RR and ECO_2_RR processes. A recent study by Zhao et al. [27] demonstrated that Cu^2+^ species present on the catalyst’s surface underwent reduction to Cu^+^ by photoinduced electrons, creating active sites that captured the in situ generated *CO intermediate and thereby facilitating the subsequent C–C coupling reaction. Nevertheless, maintaining the stability of Cu^+^ species in aqueous solutions remains a challenging problem, as it is important for prolonging the lifetime of *CO intermediate and enhancing the CO_2_ reduction into C_2+_ products. Therefore, understanding the links between the selectivity of end products and the valence state/coordination environment of copper species has been a subject of significant interest. To date, numerous electrocatalysts have been explored for the ECO_2_RR, each yielding specific reduction products. The types and the number of desired products can be adjusted by fine-tuning the binding energy of adsorbed intermediates, including *CO, *COOH, *CHO, and *COH. For example, when the interaction between the electrocatalyst surface and reduction intermediates is relatively weak, the primary products are CO and formate ions (HCOO^−^). This occurs because, in cases of weak binding, the C–O bond in these intermediates. Conversely, if electrocatalysts strongly bind *CO intermediates, the production of CO and HCOO^−^ is limited. This is because the *CO intermediate remains attached to the catalyst surface for a more extended period, allowing it to undergo further reduction into other products. Among several catalysts investigated, Cu stands out as a unique metal because it can efficiently produce C_2+_ products including hydrocarbons and alcohols [28, 29]. The basic explanation for its ability to produce C_2+_ products is that Cu binds *CO neither too weakly nor too strongly [30, 31]. Nevertheless, the selectivity of bare Cu electrodes for particular products is generally poor, leading to the simultaneous formation of a range of reduction products. On a microscopic level, the underlying reason for Cu’s poor selectivity lies in its moderate binding affinity for most reaction intermediates. Cu_2_O exhibits similar characteristics to metallic copper in terms of its ability to adsorb and activate CO_2_ that leads to the promotion of C–C bond coupling and the generation of C_2+_ chemical compounds. During the ECO_2_RR process, Cu_2_O shows rapid surface reconstruction which converts a portion of Cu^+^ to Cu^0^. Cu^0^/Cu^+^ pairs exhibit a synergistic effect that enhances the *CO adsorption on Cu_2_O surface, improving the selectivity toward C_2+_ products [32–34].

**Fig. 2:**
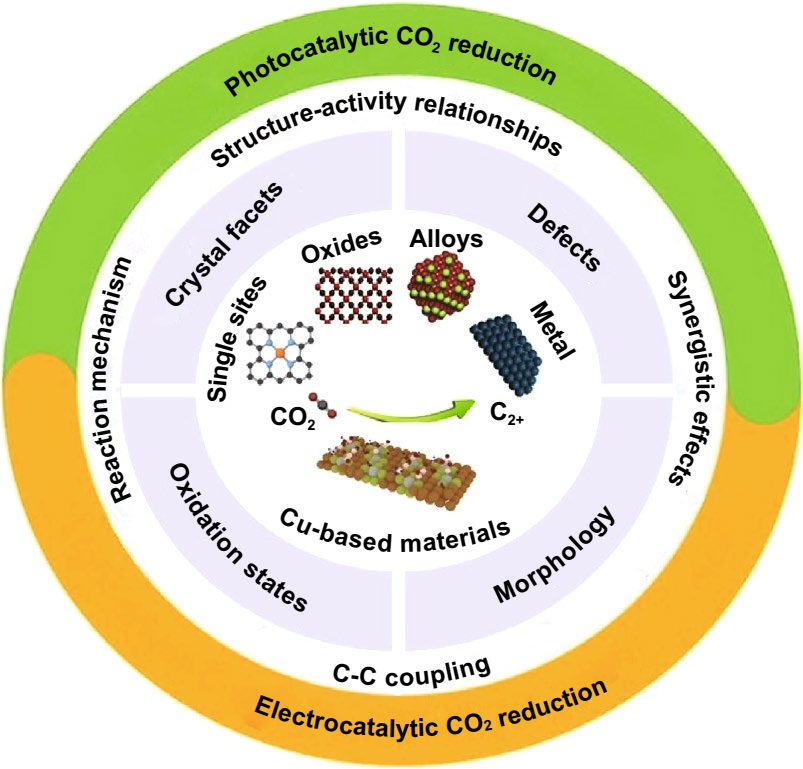
Schematic illustration of the review article

Despite the significant progress made in photocatalytic and electrocatalytic CO_2_ reduction, the factors that influence the ability of copper containing catalysts to tune the reaction mechanism and the selectivity toward C_2+_ products are not yet well understood. Herein, we report the latest advancements in Cu-based materials as catalysts for PCO_2_RR and ECO_2_RR with the aim of gaining a deeper comprehension of the structure–activity relationships. The schematic illustration of this review is shown in Fig. 2. The review begins with the fundamentals of photocatalytic and electrocatalytic CO_2_RR. In particular, we summarize the detailed mechanisms and possible reaction pathways for CO_2_ reduction to C_2+_ products on various Cu-based materials. We then introduce the application of Cu-based materials in PCO_2_RR and ECO_2_RR to C_2+_ products. To gain an in-depth knowledge of the factors that impact the catalytic performance of Cu, we have classified copper-based materials into various categories including Cu metal, Cu oxides, Cu alloys, and Cu SAs species. Finally, the main challenges to be resolved and the future perspective for Cu-based materials in converting CO_2_ into C_2+_ products are envisioned.

## Fundamental Understanding of the Mechanisms of CO_2_ Reduction Reaction

### Basic Principles of Photo- and Electro-catalytic CO_2_ Reduction

#### Photocatalytic CO_2_ Reduction Reaction

**Fig. 3:**
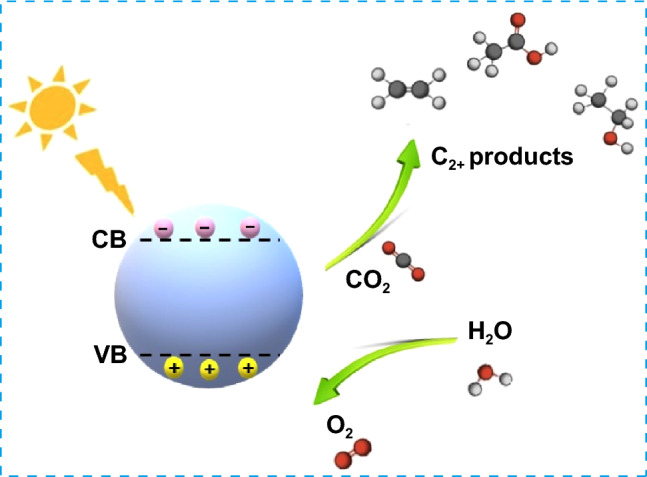
Photocatalytic CO_2_ reduction mechanism on a catalyst surface

PCO_2_RR presents a promising method for directly using solar energy to transform CO_2_ into valuable products. Photocatalytic reactors are generally configured in two ways: either using particulate semiconductor photocatalysts suspended in a CO_2_ gas-saturated solution or using uniformly distributed and immobilized photocatalysts mixed with CO_2_ gas and H_2_O vapor on a substrate. In principle, PCO_2_RR process involves three stages: absorption of light (photons), separation and transfer of charges, and subsequent reactions that take place at the interface (Fig. 3). The overall efficiency of the process is determined by multiplying the efficiency of each step. The application of PCO_2_RR process faces several challenges. In particular, the simultaneous absorption of light across a wide range of solar spectrum and carrying out oxidation–reduction reactions with a single semiconducting material is difficult. Materials with wide bandgaps, such as titanium dioxide (TiO_2_) and zinc oxide (ZnO), exhibit photoactivity within the ultraviolet (UV) spectrum, while narrow bandgap semiconductors such as Cu_2_O show activity in the visible NIR range; however, the band potentials of Cu_2_O semiconductors are not conducive to mediating both reduction and oxidation reactions simultaneously. As a result, single-component systems have lower efficiency for photocatalysis. In order to address this issue, researchers have attempted to develop heterostructures (in forms of nanowires, nanobelts, nanotubes, nanorods, etc.) [35–38]. In addition, it is crucial to achieve separation and transfer of spatial charges from the catalyst surface to the reactants for CO_2_ reduction. Nevertheless, photogenerated electrons and hole may recombine rapidly due to the coulombic attraction and the absence of charge trapping states on the surface of the catalyst, which would lower the charge separation efficiency and consequently hinder the reaction process. Therefore, a small proportion of separated charges will move toward the reactive sites located on the catalyst surface and will take part in the redox reaction, which involves the conversion of CO_2_ and H_2_O into diverse oxygenated and carbon-containing compounds. The resulting products are ultimately released from the surface of the semiconductor and separated. Apart from these issues, limitations such as insufficient capacity of active sites to adsorb CO_2_ inhibit charge accumulation and CO_2_ activation. Moreover, PCO_2_RR to C_2+_ products are hindered by the relatively slower rate of electron transfer, along with the sluggish kinetics involved in the formation of carbon–carbon (C–C) bonds, which may lead to the release of *CO from the surface of the catalyst. The release of electrons occurs before *CO can effectively accept the subsequent electrons, which are necessary for its further reduction into C_2+_ hydrocarbon products. Therefore, the attainment of efficient synthesis of C_2+_ hydrocarbons in a photocatalytic system remains a significant challenge. It was reported that Cu_2_O undergoes a preferential reduction to Cu^0^ during the photocatalytic process, serving as the dominant Cu species involved in the CO_2_ reduction reaction [39]. Furthermore, due to excellent electrical conductivity of Cu^0^, it facilitates the accumulation of photogenerated electrons, thereby increasing the electron concentration. Additionally, the higher-energy electrons generated through localized surface plasmon resonance (LSPR) of Cu can activate the typically unreactive and stable chemical bonds present in CO_2_. Nonetheless, despite the effectiveness of Cu^0^ as an active site for photocatalytic CO_2_ reduction and C–C coupling, the recombination of charge carriers on the semiconductor photocatalyst continues to hinder the accumulation of photogenerated electrons on the Cu^0^ surface. This limitation ultimately leads to relatively low photocatalytic CO_2_ reduction activity. Up to the present time, addressing this challenge and effectively bridging the gap between the lower efficiency of multielectron transfer and the sluggish kinetics associated with *CO coupling remains a persistent challenge. Researchers continue to explore and develop strategies to improve the effectiveness of charge transfer and the kinetics of C–C bond formation such as metal/non-metal doping, cocatalyst deposition, heterojunction construction, etc.

#### Electrocatalytic CO_2_ Reduction Reaction

**Fig. 4:**
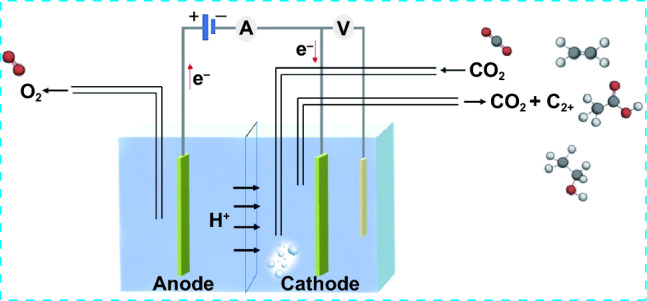
Typical electrocatalytic CO_2_ reduction system

ECO_2_RR, which can be powered by renewable energy sources, is an effective way to achieve carbon neutrality. An electrocatalytic CO_2_ reduction system typically operates in a three-electrode mode. Specifically, the working electrode and reference electrode are situated at the cathode, while the counter electrode is located at the anode. To isolate the half-reactions and enable ion migration, the two electrodes are separated by an ion exchange membrane. On the anode, H_2_O molecules are oxidized to produce oxygen and protons, with oxygen being collected as a gas product and the protons traverse the membrane to reach the cathode. The CO_2_ molecules that are present in the electrolyte are transported to the cathode surface through a combination of convection and diffusion. At the cathode, multiple steps of proton and electron transfer convert the CO_2_ into desired products through reduction potential (Fig. 4). Nevertheless, CO_2_ dissolved in the electrolytes has high ohmic and mass transfer resistance because of the distance between cathode and anode, while the cathode has limited CO_2_ diffusion due to its low solubility. An often employed and advantageous electrolyte is an alkaline aqueous solution, primarily because it exhibits a lower overpotential when compared to its neutral counterpart. When various alkaline aqueous solutions were examined, it was noted that higher current densities were achieved with increasing solution concentrations [40]. Electrochemical impedance spectroscopy further revealed a reduction in cell resistance as a result of increased ionic conductivity with rising concentrations. In their study, Sinton et al. [41] observed a notable 240-mV positive shift of the onset potential when they employed a 10 M KOH electrolyte solution instead of a 1 M KOH solution.

In general, CO_2_ solubility can be enhanced by employing organic solvents instead of aqueous solutions as electrolytes. Despite CO_2_ being a nonpolar molecule, it exhibits considerable polarizability and can form hydrogen bonds with compatible donor solvents. The majority of organic electrolytes are polar solvents, which permits electrocatalytic CO_2_ reduction over a broader potential range. In addition, using an aprotic solvent-like acetonitrile or dimethylformamide could promote the dimerization of *CO–CO, whereas the production of CH_4_ is favored in a protic solvent. There has also been research exploring the use of ionic liquids, added to either aqueous or organic solvents, which exhibit good thermal stability, high CO_2_ solubility, and high conductivity; nevertheless, their high cost and the possibility of cathodic corrosion are the challenges that should be considered.

In electrocatalytic CO_2_ reduction experiments, some researchers have introduced gaseous CO_2_ to the cathode, ensuring that an ample amount of CO_2_ is delivered to the catalyst, even when operating at high current densities. Certain research teams have achieved noteworthy enhancements in the current density for CO_2_ reduction to C_2+_ products by employing gas diffusion electrodes (GDEs) [42, 43]. Nonetheless, a notable challenge associated with GDE-type electrolyzers is their reliance on aqueous electrolytes for the collection of liquid products. The inclusion of these aqueous electrolytes can result in the dilution of the liquid products, leading to increased expenses in terms of product recovery and separation. To address the challenge posed by GDE-type reactors, several research groups have investigated the electrocatalytic CO_2_ reduction in a zero-gap type electrolyzer, which eliminates the requirement for an aqueous electrolyte [44, 45]. For example, Lee et al. introduced an effective strategy called catholyte-free electrocatalytic CO_2_ reduction (CF-CO_2_R) and designed to circumvent solubility limitations by incorporating an appropriate amount of water vapor along with gaseous CO_2_ as a cathode reactant [45]. In this CF-CO_2_R electrolyzer, water vapor serves as a carrier, facilitating the supply of dissolved CO_2_ to the cathode by creating a CO_2_-saturated aqueous film on the catalyst surface. This approach offers the advantage of replenishing consumed CO_2_ in the film directly from the bulk gas stream, thereby enhancing CO_2_ mass transfer and improving reaction kinetics. However, it is noteworthy that this configuration only permits the detection of gas products. Therefore, it is crucial to carefully choose a suitable cellular arrangement based on the properties of the catalyst and the nature of the desired products.

In general, electrocatalytic systems have a more compact and flexible structure compared to photocatalytic systems. They also have higher catalytic efficiency due to the continuous transfer of electrons to the working electrode driven by an external bias. Nevertheless, the direct redox reaction on the electrode requires a higher overpotential, resulting in relatively high-energy consumption. Additionally, adjusting the strength and form of the external potential allows for easy control of the selectivity of products derived from CO_2_ reduction. Meanwhile, the ECO_2_RR process is not as favorable in aqueous electrolytic systems when compared to competing reactions like the hydrogen evolution reaction (HER) [46]. To improve the selectivity toward C_2+_ compounds, it is necessary to supress the HER pathway and reduce the rates of C_1_ products. However, similar to the photocatalytic CO_2_ reduction systems, the electrocatalytic CO_2_ reduction mechanism is complex, and it results in the production of C_1_ hydrocarbons, leading to selectivity issues. The diverse final products arising from this complicated pathway make it challenging to obtain the desired product with good selectivity [12, 14, 47].

### The Pathway of CO_2_ Reduction to C_2+_ Products over Cu-based Materials

#### CO_2_ Adsorption/Activation and Different Models

**Fig. 5:**
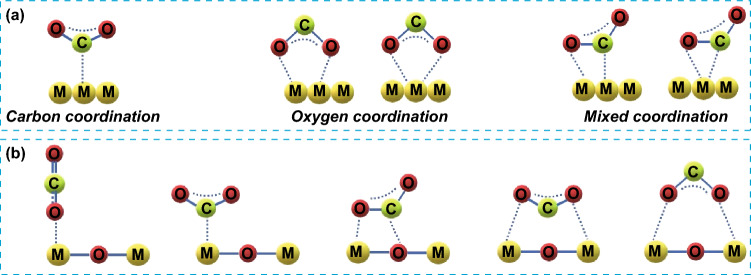
The possible configurations of adsorbed CO_2_ on: **a** metal catalysts and **b** metal oxide catalysts

To optimize the CO_2_RR process for increased C_2+_ product formation, it is essential to understand the reaction intermediates and pathways that are involved. As is well known, the CO_2_RR processes typically involve three main steps. The initial step entails the adsorption/activation of CO_2_ on the surface of the catalyst, resulting in the production of CO_2_^· -^ anion radicals (Eq. 1 in Table 1). The second involves a sequence of complex reactions that are dependent on the transfer of e^−^/H^+^, leading to the production of precursors such as *CO, *COH, and *CH_2_, or effecting C–C coupling to produce C_2+_ chemicals as listed in Table 1 (Eqs. 2–11 in Table 1). Finally, in the third step, the formed products are desorbed from the surface of the catalyst. CO_2_ is a highly stable molecule with a linear symmetry structure and its direct reduction in an aqueous solution to form CO_2_^· -^ anion radical through single-electron transfer process with a standard redox potential of − 1.9 V versus NHE is thermodynamically unfavorable. Unlike the activation of CO_2_ molecule over heterogeneous catalysts through surface atom interactions can effectively minimize the energy required to accept an electron by lowering the lowest unoccupied molecular orbital (LUMO) level of CO_2_. This can be achieved through the alteration of the molecular properties, including the elongation of C–O bond length and bending of O–C–O angle [48–51]. Generally, the CO_2_ adsorption/activation step to form CO_2_^· -^ anion radical is considered the rate-limiting step for CO_2_RR process. As of now, several models for CO_2_ adsorption on catalyst surfaces have been reported using different types of catalysts, including two main theories that focus on the use of either metals or metal oxides as catalysts [52–54]. At the molecular level, CO_2_ activation occurs through a partial transfer of electrons into the LUMO, resulting in the generation of partially charged CO_2_^δ−^ species [55, 56]. Based on metal catalysts, five configurations for CO_2_ adsorption and activation were proposed as depicted in Fig. 5a. The structure of CO_2_^δ−^ species varies according to the adsorption mode, namely, carbon coordination, oxygen coordination, and mixed (carbon/oxygen) coordination. Carbonate-like species ensue from the carbon binding mode wherein the carbon atom serves as electron acceptor for Lewis base centers. Dual bidentate species emerge through oxygen coordination, with two alternative structures. In this model, the oxygen atoms act as electron donor for surface Lewis acid centers owing to the pair electrons. Meanwhile, structures of both species occur in the mixed coordination mode. According to this model, CO_2_ molecules serve as both an electron donor and acceptor by oxygen and carbon atoms, respectively.

As for the metal oxide surfaces, CO_2_ activation can be accomplished by forming coordination bonds with adjacent metal sites, either through the carbon atom or the terminal oxygen atoms of the CO_2_ molecule, resulting in the formation of monodentate or bidentate carbonate species (Fig. 5b) [48, 57]. The first two configurations show the formation of monodentate species, in which either the oxygen or carbon atom coordinates with a metal atom of the metal oxide by forming M–O or M–C bonds. In the third configuration, the CO_2_ molecule binds to the metal oxide surface through interactions between both oxygen with metal atom and carbon with oxygen atom of the metal oxide. The fourth configuration depicts a mixed oxygen/carbon coordination, resulting in a bridged carbonate geometry, in which two oxygen atoms bind with two metal atoms, and the carbon atom of CO_2_ molecule points downward. The last configuration presents a bridging geometry through oxygen coordination, where two oxygen atoms interact with two metal atoms and the carbon atom points upward. These differed binding configurations might result in many intermediates, leading to various reaction pathways. This highlights the importance of understanding the CO_2_ adsorption/activation steps in CO_2_RR processes.

**Fig. 6:**
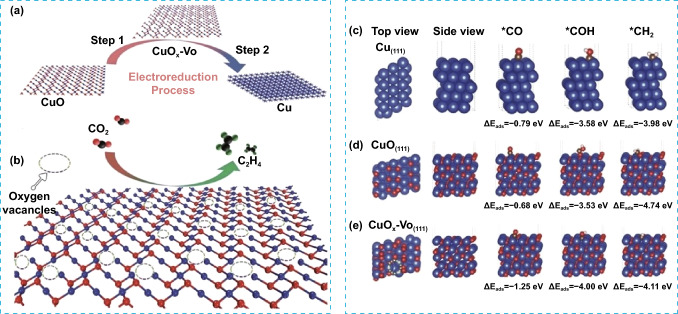
**a** V_O_-rich CuO_*x*_ surface via electrochemical control of oxygen vacancies. **b** Schematic illustration of ECO_2_RR into C_2_H_4_ on Vo-rich CuO_*x*_–Vo surface. DFT calculation results with three ECO_2_RR intermediates (*CO, *COH, and *CH_2_) of** c** pure Cu, **d** Vo-free CuO, and **e** CuO_*x*_–Vo. Reproduced with permission [67]. Copyright 2018, Wiley–VCH

The surface structure of metal oxides plays a key role in CO_2_ adsorption/activation process. Different structures may have different active sites and surface energies that can affect the strength of the interaction between CO_2_ molecules and metal oxides. As example, the CO_2_ adsorption on CuO oxide surfaces, namely, (0 1 1), (1 1 1), and (− 1 1 1), was notably strong only on the (0 1 1) surface. Conversely, a weak CO_2_ adsorption was observed on CuO (1 1 1) and CuO (− 1 1 1) surfaces [58]. As reported in the literature, oxygen vacancies Vo could change the physico-chemical and electronic properties of metal oxides such as TiO_2_ [59–61], CeO_2_ [62], In_2_O_3_ [63], Zn_2_GeO_4_ [64], and Cu_2_O [65, 66] leading to improved surface adsorption and the creation of additional active centers. Zheng and co-workers reported that copper oxide nanodendrites with partially reduced surfaces and abundant Vo (CuO_*x*_–Vo) exhibited improved CO_2_ adsorption and electroreduction abilities when compared to pure Cu and Vo-free CuO_*x*_ (Fig. 6a and b). The surface Vo has been identified as effective Lewis base sites for the enhancement of CO_2_ adsorption. Theoretical calculations showed that CuO_*x*_–Vo provides strong binding affinities toward *COH and *CO intermediates, while displaying weak affinity toward *CH_2_, resulting in efficient production of C_2_H_4_ with high Faradaic efficiencies reaching 63% (Fig. 6c–e). Moreover, it was demonstrated that the Faradaic efficiency for C_2_H_4_ production is greatly influenced by the density of Vo in CuO_*x*_ [67]. This highlighting the potential of controlling and engineering Vo defects to create more effective catalytic materials for CO_2_ adsorption/activation and to adjust the selectivity toward desired products in CO_2_RR. Aside from surface defects, the deposition of basic sites such as alkaline or alkali-earth metals on the catalyst surface can promote the CO_2_ adsorption owing to the strong interaction with acidic CO_2_ molecules. Also, increasing the catalyst’s surface area could provide more active sites for CO_2_ adsorption [68]. Furthermore, surface modification involving the addition of functional groups, such as hydroxyl (OH) or amino (NH_2_) groups, has been observed to exert significant effects on the interaction between CO_2_ and the surface, thereby improving its adsorption [69, 70].

#### The Pathway of CO_2_ Reduction to C_2+_ Products

**Fig. 7:**
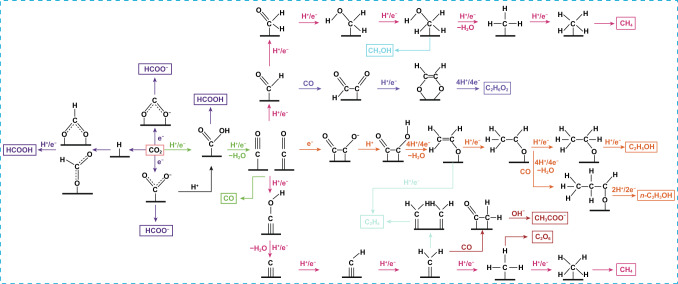
General pathways for CO_2_ reduction reactions. Reproduced with permission [22]. Copyright 2023, Wiley–VCH

As illustrated in Table 1, the CO_2_RR process includes a complex reaction mechanism, which gives rise to the production of multiple products. Consequently, obtaining the desired product becomes challenging due to selectivity concerns. The determination of the favored reaction pathway leading to the wanted product is intricately dependent on the adsorption energy of the intermediates at the active site of the catalyst. As an example, the formation of HCOOH from the key intermediate *OCHO or *COOH depends on the type of adsorption model type resulted from the hydrogenation of the CO_2_^· -^ anion radical adsorbed onto the catalyst surface [71]. As shown in Fig. 5, *COOH is also a key intermediate in the generation of *CO, which subsequently undergoes desorption to yield the gaseous product CO [72]. The formation of C_2+_ chemicals requires the exchange of a greater number of electrons in comparison with C_1_ products, and the subsequent coupling of C–C bonds is deemed to be the step that governs the reaction rate, making this process difficult to occur kinetically. *CO is a key intermediate as it plays a significant role in the C–C coupling reaction, which leads to the generation of C_2+_ products [7, 73]. It has been reported that *COCHO plays a crucial role as an intermediate in the production of C_2+_ products, which may be formed through various coupling reactions, such as the coupling of CO and *CHO (*CHO + CO → *COCHO), the coupling of CO and *COH (*COH + CO → *COCOH), or coupling *CO (*COCO + H^+^  → *COCHO) [73, 74]. Compared to *COCOH, the *COCHO intermediate is more stable as it lacks a double bond to the active site or a free radical on the carbon atom. As illustrated in Fig. 7, the ethylene pathway involves the hydrogenation of *COCHO to *COCHOH, followed by its conversion to *OCH_2_COH. This ultimately yields both and ethylene (C_2_H_4_) and acetic acid (CH_3_COOH). In the ethanol pathway, the reaction mechanism involves the conversion of *COCHO to glyoxal (C_2_H_2_O_2_) through a one-step hydrogenation process. This glyoxal can be further transformed into either acetaldehyde (CH_3_CHO) or ethanol (CH_3_CH_2_OH) based on the potential applied to the reaction. A lower potential favors the production of acetaldehyde, whereas at higher potential, the reaction yields ethanol [74, 75]. Furthermore, it is possible to form ethylene, ethane, ethanol, and acetaldehyde products through the carbene route, which entails the generation of CO* as a major intermediate compound. The CO intermediate is subsequently fully reduced to “C” and further reduced to form CH_2_^·^ and CH_3_^·^ radicals. If the photocatalyst surface can stabilize the CH_2_^·^ and CH_3_^·^ radicals, then they are more likely to couple and form C_2+_ products. However, if the surface cannot stabilize these radicals, then they will desorb as methane.

A reaction pathway involving the coupling of three carbon atoms is required for the production of C_3_ products. García et al. utilized DFT calculations to conduct an in-depth analysis of the mechanism involved in CO_2_ conversion to C_3_ products. According to their study, C_3_ products are formed by combining C_2_ and C_1_ intermediates [76]. These interactions might occur between C_1_ (CH_*x*_O*, CH_x_*), and C_2_ intermediates, which may include hydrocarbons (CH_*y*_CH_z_*) or oxygenates (CH_*y*_OCH_*z*_O*, CH_*y*_OCH_z_*, and CH_*y*_CH_*z*_O*). However, the current selectivity for C_3_ products is still low. Thus, further investigations of the reaction pathways are needed design effective catalysts that can promote the formation of C_3_ products.

While numerous reaction pathways have been confirmed by theoretical and experimental approaches, the actual CO_2_RR processes are influenced by many factors, and the products selectivity is more complex. In the case of PCO_2_RR process, the selectivity to C_2+_ is particularly intricate and largely influenced by the photoinduced e^−^/h^+^ density as well as the stability of the generated intermediates. The C_1_ products are rapidly generated as the intermediate can easily combine with hydrogen atoms, while the opposite charge of intermediates can obstruct the C–C coupling steps. On the other hand, several factors significantly influence the ECO_2_RR process’s selectivity, including operating conditions, which consist of the electrolyte pH, the type of electrolyte cation/anion, and the applied overpotential [74]. In addition, both systems can be influenced by the catalyst surface properties. These properties, including surface adsorbates, defects, structure, morphology, and facets, can substantially alter the adsorption energies of critical intermediates as well as the kinetic barriers of reactions, resulting in different reaction routes [77]. Despite the fact that PCO_2_RR and ECO_2_RR have certain distinctions, their practical implementation is hampered by comparable hurdles and comparable strategies aimed at increasing the efficiency and the C_2+_ products selectivity as well as the stability may be used in both systems to overcome the challenges.

## Fine-Tuning Surface Structure of Cu-Based Catalysts for Improving the Activity/Selectivity of CO_2_RR Toward C_2+_ Compounds

### Photocatalytic CO_2_ Reduction Reaction

Despite the potential of PCO_2_RR as a promising and sustainable approach to synthesize C_2+_ compounds, the current literature points out a relatively low efficiency and selectivity of this process in C_2+_ product formation [78, 79]. This is due to limitations on light absorption, rapid charge carriers recombination that results in low electron concentrations on the photocatalyst’s surface, high kinetic barriers, as well as desorption of C_1_ intermediates. It is well known that Cu-based photocatalysts are effective at multi-electron transfer and can make use of weakly bound *d*-band electrons. They also have a short bandgap, allowing them produce sufficient charges through the absorption of sunlight within the range of visible light. Moreover, their strong CO_2_ adsorption and their ability to effectively stabilize the reaction intermediates contribute to their potential for C_2+_ product generation. Nonetheless, the development of Cu-based photocatalysts that are highly efficient and selective in generating C_2+_ products remains a significant challenge. Researchers have been exploring different approaches such as crystal phase/morphology optimization, metal doping, defect engineering, heterostructure fabrication, and bimetallic synergies to enhance the activity/selectivity of PCO_2_RR toward C_2+_ compounds; nevertheless, there is still debate surrounding the origin of C_2+_ selectivity enhancement. In this section, we provide basic comprehension and discussions on these strategies to help with further enhancement of the activity/selectivity toward C_2+_ compounds in PCO_2_RR. To attain a more comprehensive understanding of the structure–activity–selectivity relationships, we have categorized Cu-based photocatalysts into four groups: Cu oxides/sulfides, Cu alloys, Cu-based single-atom catalysts (Cu SACs), and Cu-based heterojunctions.

#### Cu Oxides/Sulfides

**Fig. 8:**
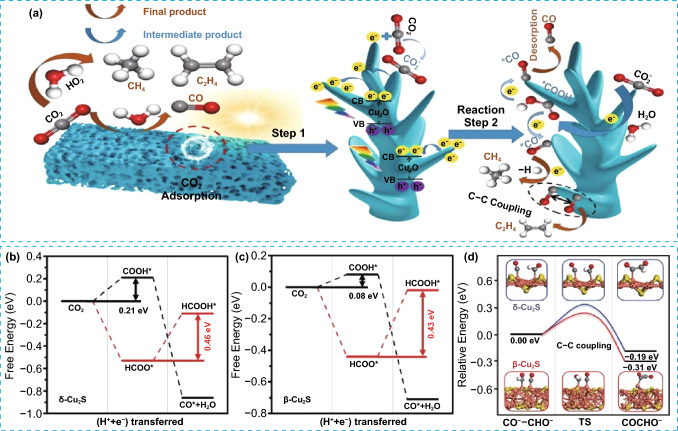
**a** Schematic illustration of CO_2_ photoreduction mechanism on 3D porous Cu_2_O. Reproduced with permission [84]. Copyright 2022, Elsevier. The free energy profiles of CO_2_ reduction to CO* and HCOOH* on **b** δ-Cu_2_S, **c** β-Cu_2_S and **d** relative energy diagram of the CHO*–CO* coupling on δ-Cu_2_S and β-Cu_2_S. Cu, orange; S, yellow; C, gray; O, red; and H, white. Reproduced with permission [85]. Copyright 2021, The Royal Society of Chemistry

Copper oxide photocatalysts are *p*-type semiconductors with narrow bandgap energy and elevated conduction band values enabling them to convert CO_2_ into various hydrocarbons [80–82]. However, the high charge recombination rate and the poor stability of Cu oxides result in a low efficiency of photocatalytic CO_2_ reduction [83]. The nano-level structural modification has been widely adopted for Cu oxides to overcome these drawbacks. Xue et al. prepared a dendritic 3D porous Cu_2_O structure via a method involving electrodeposition as well as a subsequent thermal oxidation [84]. The findings indicated that the 3D porous Cu_2_O exhibited highly effective photocatalytic performance, with a CO yield of 26.8 μmol g^−1^ h^−1^, which was 24 times greater than the CO yield obtained from the non-porous Cu_2_O structure. The 3D porous Cu_2_O with nano-sized dendrite structure was demonstrated to promote the separation of charge carriers and transport efficiency as well as the overall mass transfer efficiency of CO_2_ gas, boosting the photoreduction of CO_2_ and the anti-photocorrosion properties. Importantly, CH_4_ and C_2_H_4_ products were observed for the 3D porous structure, owing to longer retention time of gas adsorption, as well as abundant active sites and high electron transfer rate offered by the porous structure. The conversion pathways of CO_2_ to CO, CH_4_, and C_2_H_4_ products are depicted in Fig. 8a. The formation of *COOH intermediates occurred initially from CO_2_ reduction, which further resulted in the generation of *CO intermediates upon dehydration. The *CO intermediates desorb rapidly to generate CO products, while longer *CO adsorption time favors the production of CH_4_ and C_2_H_4_ compounds. Thus, the distribution of the final product depends on the stability of the *CO intermediate. The absorption of *CO intermediates was facilitated by the presence of Cu^+^ species, which further undergo C–C coupling steps to produce C_2_H_4_ product. This work provided insight for studying the morphology control effect for PCO_2_RR.

Another strategy for adjusting the C_2+_ product selectivity of CO_2_ reduction is crystal phase control. Recently, Wang et al. demonstrated through DFT calculations that the conversion of CO_2_ into ethanol (C_2_H_5_OH) can be efficiently achieved by both 2D β-phase Cu_2_S bilayer and δ-phase Cu_2_S monolayers [85]. Figure 8b and c illustrates the free energy profiles of CO_2_ reduction on δ-Cu_2_S and β-Cu_2_S. Two different pathways were examined, namely, CO_2_–COOH*–CO* and CO_2_–HCOO*–HCOOH*. Based on the results, the CO* intermediate was more likely to be stabilized on both surfaces. This occurred because the CO_2_–COOH* route has a lower energy barrier than the HCOO*–HCOOH* pathway. Specifically, the energy barrier for CO_2_–COOH* pathway was 0.21 eV on δ-Cu_2_S and 0.08 eV on β-Cu_2_S, while the energy barrier for HCOO*–HCOOH* pathway was 0.46 eV on δ-Cu_2_S and 0.43 eV on β-Cu_2_S. The results of Bader charge analysis show a significant charge transfer between Cu and S atoms on the surface resulting in the generation of Cu^+^ sites. This led to the adsorption of CO* and the subsequent coupling of CO* and formyl (CHO*) species, as illustrated in Fig. 8d. Notably, the kinetic barriers associated with CO*–CHO* coupling have been determined to be 0.3 eV, confirming the possibility of CO_2_ to C_2_H_5_OH conversion. These findings offer important theoretical insights for future experimental development and synthesis of photocatalysts aiming at producing C_2+_ products from CO_2_.

#### Cu Alloys

Recent studies have reported that Cu alloys have superior photocatalytic properties compared to single Cu catalysts [15, 37, 86–89]. The use of Cu alloys can improve the CO_2_ activation and optimize the binding strength of the key intermediates on catalysts surface, which ultimately promotes the efficient formation of C_2+_ chemicals via C–C coupling. Therefore, by altering the catalyst composition, the reaction mechanism can be fine-tuned and tailored to C_2+_ production. For instance, Shankar et al. synthesized Pt–Cu alloys supported on TiO_2_ nanotubes for photocatalytic CO_2_ reduction. The hydrocarbon production rate was maximized over Cu_0.33_–Pt_0.67_/TiO_2_ photocatalyst, and CH_4_, C_2_H_4_, and C_2_H_6_ products were obtained at rates of 2.60, 0.33, and 0.47 mL g^−1^ h^−1^, respectively. Whereas, the monometallic Cu/TiO_2_ or Pt/TiO_2_ catalysts and the other Cu–Pt compositions resulted in limited C_2+_ formation, with CH_4_ being the major reaction product [37]. This is possibly attributed to the ability of Pt to boost the photocatalytic reduction rates and the role of Cu in promoting the C_2+_ products selectivity owing to the higher reactivity and strong adsorption of CO on copper surface. This finding underscores the importance of carefully controlling the composition of the photocatalyst to tune the selectivity toward C_2+_ products. In et al. observed a shift in selectivity from CH_4_ to C_2_H_6_ by CO_2_ photoreduction under artificial sunlight (AM1.5) using bimetallic Cu–Pt alloys deposited reduced blue titania (Cu–Pt/BT) catalysts. The enhanced C_2_H_6_ selectivity was attributed to the effective transfer of high density of electrons from BT to Cu nanoparticles through Pt, along with the high concentration of stabilized CH_3_^·^ intermediates [78]. The formation of C_2_H_6_ product occurs through the reaction of two CH_3_^·^ radicals via a self-reaction process.

Apart from the previously mentioned limitations regarding the production of C_2+_ compounds during photocatalytic CO_2_ reduction, the repulsion between reaction intermediates generated during the process also impede C–C coupling reaction required for the generation of C_2+_ compounds. To minimize the inter-adsorbate repulsive forces, one can establish nearby reaction sites with opposing charges. In this regard, Shankar et al. synthesized large-sized AgCu nanoparticles supported on TiO_2_ nanotube array (AgCu-TNTA) [15]. The obtained photocatalyst exhibited total rate of hydrocarbon production (CH_4_ + C_2_H_6_) of 23.88 μmol g^−1^ h^−1^ with C_2_H_6_ selectivity of 60.7%. In comparison, the Ag-TNTA and Cu-TNTA catalysts showed C_2_H_6_ selectivity of 15.9% and 10%, respectively, indicating that the presence of both Ag and Cu in the AgCu bimetallic alloy resulted in a synergistic effect, enhancing the production of C_2_H_6_ in PCO_2_RR process. The synergistic effect was related to the multipolar resonances in large plasmonic AgCu nanoparticles, which enabled the creation of active sites with opposite charges, thus reducing the repulsion between reaction intermediates. Additionally, the stabilization of CH_3_^·^ radicals was observed due to the ability of both Ag and Cu to stabilize radicals through the promotion of C–C coupling on their surfaces. This occurred through charge transfer, where plasmonic electrons were injected into the TNTAs, leaving behind holes that gave the metals a positive charge. This positive charge increased the lifespan of CH_3_^·^ radicals. Moreover, the researchers noted that a high concentration of hot spots, where the electric field is particularly strong, may increase the polarization of CO_2_ molecules and promote the production of C_2+_ compounds.

**Fig. 9:**
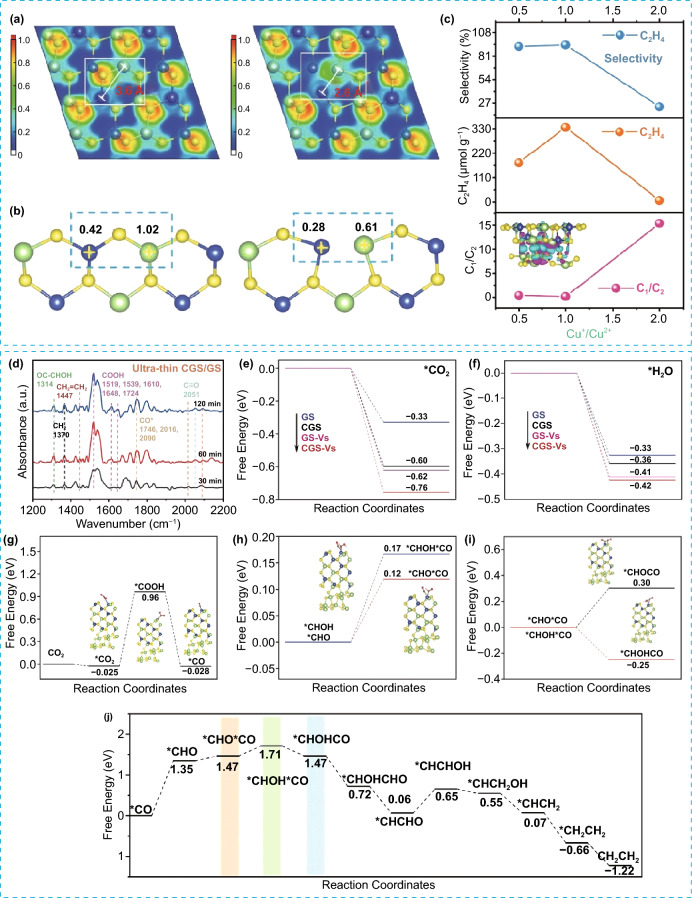
**a** ELF of CGS/GS (Left) and ultra-thin CGS/GS with S vacancy (Right). **b** The calculated Bader charge of CGS/GS (Left) and ultra-thin CGS/GS with S vacancy (Right). **c** The correlation of C_1_/C_2_, C_2_H_4_, and selectivity with Cu^+^/Cu^2+^, **d** in situ FTIR spectra on ultra-thin CGS/GS. **e** Free energy of CO_2_ on different active sites. **f** Free energy of H_2_O on different active sites. **g** Free energy of CO_2_ photoreduction to CO. **h**, **i** Free energy of different intermediates. **j** Schematic diagram of CO_2_RR. Reproduced with permission [93]. Copyright 2023, Wiley–VCH

Numerous studies reported that defect engineering played an important role in governing both the interface electronic structure and active sites of catalysts. This, in turn, can significantly impact the photocatalytic process [90–92]. For instance, Yu et al. fabricated a 2D ultra-thin CuGaS_2_/Ga_2_S_3_ (CGS/GS) with S vacancy, which showed unprecedented selectivity toward C_2_H_4_ (≈ 93.87%) with a production rate of 335.67 µmol g^−1^ h^−1^ [93]. They found that the mechanism for the multi proton–electron pathway of CO_2_ reduction reaction is altered by the existence of S vacancy. This occurs because S vacancy triggers a highly delocalized electron distribution, leading to a local metallization between Cu and Ga in the vicinity of the S vacancy and resulting in the formation of Cu–Ga metallic bond (Fig. 9a and b). These bimetallic Cu–Ga dual sites could facilitate the C–C coupling and stabilize the formed intermediates, thereby lowering the energy barrier for C_2_H_4_ formation. Also, they noted that the photocatalysts’ selectivity is dependent on the Cu oxidation state. As shown in Fig. 9c, the increase in Cu^+^/Cu^2+^ ratio resulted in an increase in C_2_H_4_ yield and selectivity owing to the improved thermodynamics of *CO dimerization by Cu^+^ species. However, when the Cu^+^/Cu^2+^ ratio exceeded 2, the yield and selectivity of C_2_H_4_ decreased significantly, which was attributed to insufficient Cu^2+^, resulting in a notable reduction in the adsorption capacity of *CO intermediate. In other words, the catalyst surface underwent a charge distribution rearrangement as a result of introducing S vacancies, which dominantly affects the chemical state of Cu ions. These effects are likely to be beneficial for the production of C_2+_ compounds. The reaction mechanism has been investigated by in situ Fourier transform infrared spectroscopy (FTIR). Figure 9d shows the presence of signals relative to *COOH, *CO, and *OCCHOH intermediates, indicating that *OCCHOH was a key intermediate of coupling of C–C for C_2_H_4_ formation. The DFT results reveal that *COOH intermediates were generated from CO_2_ reduction, which subsequently underwent coupling with H^+^/e^−^ pairs to yield CO molecules. Eventually, *CO intermediates transform into C_2_H_4_ through complex processes involving electron and proton transfer (Fig. 9e–i). In addition, the formation of *CHOHCO by coupling *CO and *CHOH appears to be the most thermodynamically favorable pathway for C–C bonding formation when compared to other pathways (Fig. 9j). This study uncovered a novel approach to optimize the geometric distance of reactive sites through vacancy engineering for increasing the efficiency/selectivity of CO_2_ photoreduction into C_2+_ products.

#### Cu SACs

Single-atom catalysts (SACs) have become a prominent area of research as they allow the utilization of almost all active metal sites [94–97]. To achieve this, metal particles are reduced in size and aggregation to form individual atoms with low coordination states. This leads to single-atom SAs having exclusive electronic properties that distinguish them from corresponding bulk materials [98, 99]. For PCO_2_RR, metal SAs doping into substrates has been studied extensively to alter photocatalysts properties. Metal ions can act as hole trappers which, in turn, facilitate water oxidation and produce a significant amount of protons that can form specific C_1_ intermediates. This can promote the C–C coupling and help in regulating the selectivity toward C_2+_ compounds by altering pathway the CO_2_ photoreduction pathway [100–107].

For instance, Cu-doped semiconductors such as TiO_2_ and g-C_3_N_4_ have been employed as catalysts for PCO_2_RR. Huang et al. synthesized a catalyst composed of Cu–N_4_ sites supported by phosphorus-modulated g-C_3_N_4_, denoted as CuACs/PCN. The CuACs/PCN photocatalyst exhibited high efficiency in producing C_2_H_4_, demonstrating a selectivity of 53.2% and a yield rate of 30.51 µmol g^−1^. Experimental and theoretical investigations revealed that C–C coupling intermediates could be formed on Cu–N_4_ sites, and the presence of P in the surrounding microenvironment of CuACs/PCN lowered the energy levels of intermediate reactions [100]. It was found that CuACs/PCN exhibited lower energy barrier in almost all of the stepwise reactions, indicating the significant role played by P in enhancing C_2_H_4_ product selectivity. This research study emphasizes the importance of fine-tuning the coordination environment and the surrounding microenvironment of Cu SAs for the efficient formation of C_2_H_4_. Moreover, it provides a promising strategy that could be utilized to achieve selective C_2_H_4_ production in photocatalytic systems. Wang et al. conducted a study where they integrated Cu SAs into a UiO-66-NH_2_ support for PCO_2_RR [101]. They found that the Cu SAs and UiO-66-NH_2_ interact through the –NH_2_ groups. The resulting catalyst has the ability to transform CO_2_ into liquid-phase products, including CH_3_OH at a rate of 5.33 µmol h^−1^ g^−1^ and C_2_H_5_OH at a rate of 4.22 µmol h^−1^ g^−1^. According to DFT calculations, the integration of Cu SAs resulted in a downshift of the HOMO and LUMO energy levels of UiO-66-NH_2_, resulting in a narrowed bandgap of the catalyst. Moreover, the Fermi level of Cu/UiO-66-NH_2_ was lower than the LUMO level of UiO-66-NH_2_, which enhances the electron transfer efficiency. By comparing the partial density of states (PDOS) of UiO-66-NH_2_ and Cu/UiO-66-NH_2_, it was found that the introduction of Cu single atoms can decrease the bandgap of UiO-66-NH_2_ by shifting its CBM to the Fermi level, which explain the C_2+_ products formation over Cu/UiO-66-NH_2_.

**Fig. 10:**
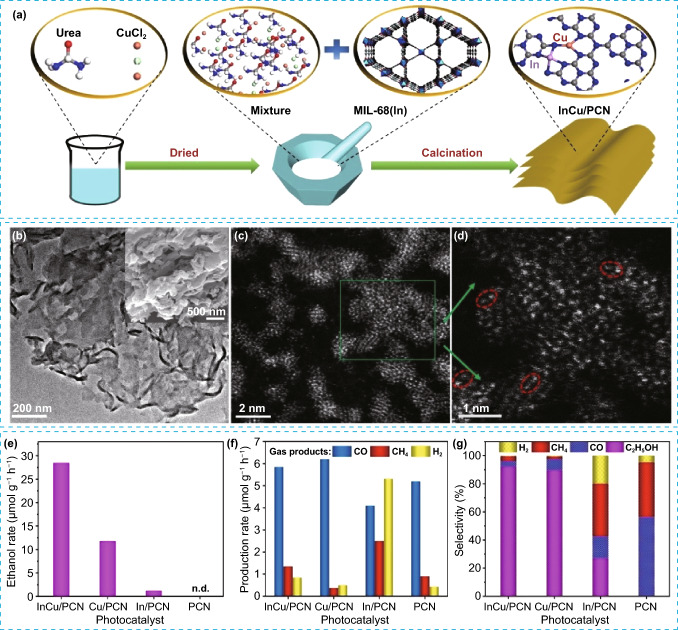
**a** Schematic illustration of the synthetic process of InCu/PCN, **b** TEM image with SEM image inset of InCu/PCN sample, **c**, **d** AC-HAADF-STEM images of InCu/PCN, CO_2_ photoreduction over InCu/PCN, Cu/PCN, In/PCN, and PCN, **e** ethanol production rate, **f** gas products generation rate, and **g** product selectivity. Reproduced with permission [104]. Copyright 2022, Wiley–VCH

Recent research suggested that bimetallic SACs exhibit superior photocatalytic performance compared to monometallic SACs for PCO_2_RR. This is can be attributed to the synergistic effect between the two metals, which promotes the activation of CO_2_ and stabilizes the reaction intermediates, thereby promoting the C–C coupling process that ultimately leads to the generation of C_2+_ products [102–104]. In this regard, Huang et al. developed a tandem photocatalysis strategy by combining rhenium-(I) bipyridine fac-[Re^I^(bpy)(CO)_3_Cl] (Re-bpy) and copper-porphyrinic triazine framework [PTF(Cu)] to create synergistic dual sites [91]. Under visible light irradiation, this approach effectively generated C_2_H_4_ at a rate of 73.2 μmol g^−1^ h^−1^. However, using only Re-bpy or PTF(Cu) catalysts resulted in generation of CO under similar conditions, and C_2_H_4_ could not be obtained. The tandem photocatalytic system allowed for the CO produced at the Re-bpy sites to be adsorbed by neighboring Cu SAs in PTF(Cu), leading to a synergistic C–C coupling process that resulted in C_2_H_4_ formation. Liu et al. used MIL-125(Ti) metal organic framework as a precursor and template to create a cake like porous TiO_2_with doping of Cu and Co. Results showed that after 3 h of simulated sunlight irradiation in water vapor, CO and CH_4_ were the main products for both pure TiO_2_ and 1%Cu/TiO_2_ photocatalysts [103]. The activity of 1%Cu/TiO_2_ catalyst was observed to be superior to that of pure TiO_2_, which can be ascribed to the reduction in the bandgap, thereby facilitating the separation of photoinduced charge carriers. Meanwhile, the introduction of trace Co ions through doping resulted in a shift of main products from CO and CH_4_ to C_2_H_6_, with a small amount of C_3_H_8_ also detected. The results showed CH_3_^·^ radical enrichment over Co–Cu/TiO_2_ in comparison with Cu/TiO_2_ sample. Thus, doping with Co ions led to a substantial enhancement in the selectivity of C_2+_ products. It is noteworthy that the Co/TiO_2_ photocatalyst did not produce any C_2_H_6_, indicating that the cooping of Cu and Co in the catalyst was responsible for the formation of C_2+_ products. Guo et al. conducted a study wherein they synthesized a photocatalyst composed of polymeric carbon nitride anchored with atomically dispersed Cu and In metals, referred to as InCu/PCN. The samples were synthesized by thermal polymerization method starting from mixed “CuCl_2_ + urea + In-MOF” precursor as illustrated in Fig. 10a [104]. The findings from the SEM and STEM analyses indicated that the InCu/PCN composed of Cu and In atoms that are uniformly dispersed at the atomic level on PCN nanosheets. Additionally, the results revealed the presence of distinctively paired In-Cu configurations (Fig. 10b–d). Interestingly, a remarkable ethanol production rate of 28.5 μmol g^−1^ h^−1^ with a high selectivity of 92% was achieved by InCu/PCN photocatalyst, which is 2.4 times greater than that by Cu/PCN (Fig. 10e–g). The theoretical and experimental results indicated that the interaction between In and Cu serves to improve the separation of charges by accelerating the transfer of charges from PCN to the metallic sites. Additionally, the existence of Cu–N–In bonds allowed In to transfer electrons to Cu sites, resulting in a higher electron density at the copper active sites. The interaction between Cu and In atoms also enhanced the adsorption of *CO intermediates and reduced the energy required for C–C coupling. The improved selectivity toward C_2+_ products in CO_2_ reduction reaction can be attributed to the synergistic effects of In–Cu dual-metal sites.

**Fig. 11:**
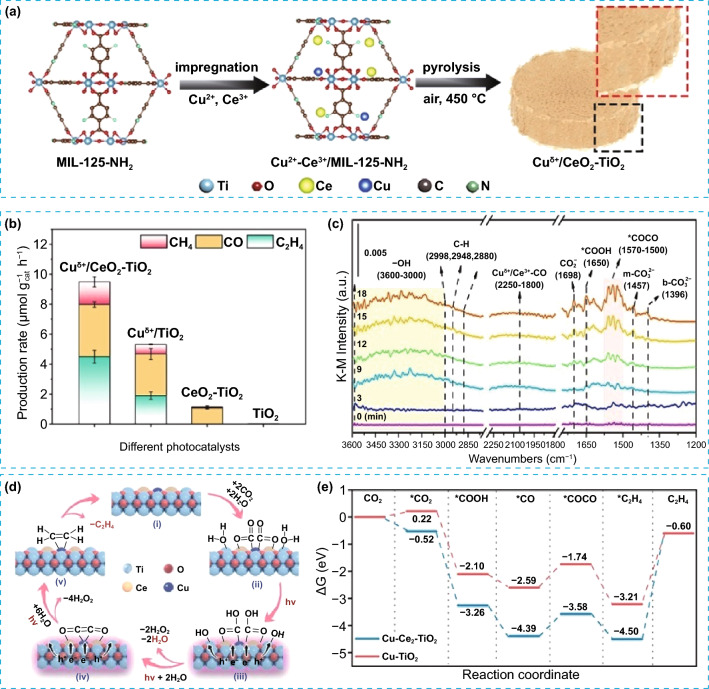
**a** Schematic illustration of the steps for Cu^δ+^/CeO_2_-TiO_2_ preparation, **b** production rates over different photocatalysts, **c** in situ DRIFTS spectra of Cu^δ+^/CeO_2_–TiO_2_ photocatalyst, **d** proposed reaction pathway for CO_2_ photoreduction to C_2_H_4_, and **e** calculated free energy diagrams for CO_2_ reduction over Cu-TiO_2_ and Cu–Ce_2_–TiO_2_ slabs. Reproduced with permission [105]. Copyright 2022, American Chemical Society

The strong metal–support interaction can enhance the dispersity and stability of SAs, thus leading to better performance of photocatalysis [108–110]. With this thought in view, Li et al. have synthesized Cu^δ+^ sites atomically dispersed on a CeO_2_–TiO_2_ support comprising of widely dispersed CeO_2_ nanoparticles on a porous TiO_2_ substrate via the pyrolysis of a metal–organic framework (MIL-125-NH_2_) impregnated with Cu^2+^ and Ce^3+^ ions (Fig. 11a). The obtained catalyst displayed increased activity toward CO_2_ reduction to C_2_H_4_ under simulated sunlight with production rate of 4.51 μmol g^−1^ h^−1^ and 47.5% selectivity, which are 2.36 and 1.32 times those over the Cu^δ+^/TiO_2_ sample (Fig. 11b) [105]. In situ FTIR presented the characteristic spectral peaks of *COOH, *CO, and *COCO groups (Fig. 11c), suggesting that the CO_2_ reduction pathway for producing C_2_H_4_ on Cu^δ+^/CeO_2_–TiO_2_ photocatalyst involved CO_2_ → *CO_2_ → *COOH → *CO → *COCO → C_2_H_4_ (Fig. 11d). In addition, according to the Gibbs free energy calculations, the whole process of CO_2_-to-C_2_H_4_ conversion on Cu^δ+^/CeO_2_–TiO_2_ catalyst is more thermodynamically favorable than on Cu/TiO_2_, as shown in Fig. 11e. This is due to the presence of Cu–Ce dual active sites, which enables the efficient generation of key intermediate *CO and support the *CO → *COCO coupling reaction. This demonstrated a synergistic effect between the active sites of Cu and Ce, which optimize the rate-limiting steps and enhance the overall CO_2_-to-C_2_H_4_ conversion. More recently, Wang et al. conducted a study exploring how the valence state and coordination environment of SAs active sites affect the C_2+_ products selectivity using 2D WO_3_ catalyst modified by depositing SAs Cu and Pt (CuPt/WO_3_). The CuPt/WO_3_ photocatalyst was found to be much more efficient than pristine 2D WO_3_, Cu/WO_3_, and Pt/WO_3_ in producing acetic acid (CH_3_COOH), with a production rate of 19.41 μmol g^−1^ h^−1^ and a selectivity of 88.1%. It has been demonstrated that stabilization of Cu^+^ species by forming a coordinated complex with Cl in aqueous solution is the key to attain superior efficiency and selectivity toward C_2+_ products [106]. All the components in CuPt/WO_3_ photocatalyst work synergistically toward the production of C_2+_ products. Cu^+^ species coordinated with Cl enhanced the CO adsorption capacity and increased the lifespan of CO* intermediate, both of which aid in the C–C coupling reaction. On the other side, SAs Pt active sites located in close proximity provided protons for the hydrogenation of CO* intermediate, ultimately leading to the formation of C_2+_ products.

**Fig. 12:**
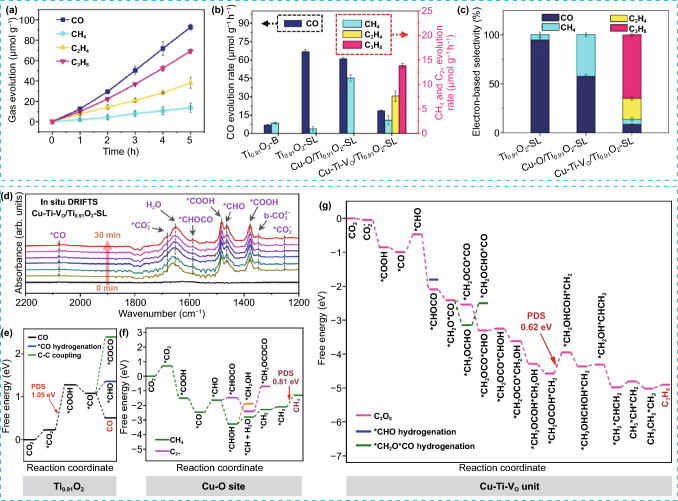
**a** The evolution of photocatalytic production as a function of light irradiation times on Cu–Ti–V_O_/Ti_0.91_O_2_–SL, **b** production rates, **c** electron-based selectivity, **d** in situ DRIFTS spectra of CO_2_ reduction on Cu-Ti-V_O_/Ti_0.91_O_2_-SL photocatalyst, gibbs free energy diagrams of CO_2_ reduction on **e** Ti_0.91_O_2_ matrix, **f** Cu–O site, and **g** Cu–Ti–VO unit. Reproduced with permission [107]. Copyright 2023, Nature

Based on the amount of studies published on photocatalytic CO_2_ reduction to C_2_ vs C_3_ products, it is obvious that direct conversion of CO_2_ into C_3_ products is much more difficult [107, 111]. This is due to the fact that the process requires a higher-order reaction pathway involving the creation of multiple C–C bonds. This, in turn, necessitates the integration of two distinct catalytic steps of CO_2_-to-CO and CO-to-C_2+_ at different active sites [112–114]. Creating these C–C bonds is particularly challenging as it involves energy-intensive endothermic reactions with significant uphill energy barriers, primarily due to the high-energy levels of the critical *C_2_ and *C_3_ intermediates. This is mainly due to the lack of efficient catalytic active sites to stabilize these intermediates and reduce energy barriers. To address this concern, Xiong et al. recently developed an efficient photocatalyst for the direct CO_2_ conversion into C_3_H_8_ [107]. The catalyst consisting of Cu SAs implanted on Ti_0.91_O_2_ atomically thin single layers (Cu–Ti–V_O_/Ti_0.91_O_2_-SL), which showed superior efficiency toward CO_2_ photoreduction to C_2+_ products with high selectivity of 50.2% for C_2+_ products and 32.4% for C_3_H_8_ as shown in Fig. 12a–c. As a comparative experiment, on Cu–O/Ti_0.91_O_2_–SL without V_O_ only showed the detection of CO and CH_4_, while Ti_0.91_O_2_–SL showed mainly CO_2_ reduction to CO product. Notably, the presence of V_O_ resulted in a strong coordination interaction between Cu SAs and neighboring Ti atoms, leading to high electron accumulation at copper sites and electron depletion at Ti sites. Conversely, Cu–O/Ti_0.91_O_2_–SL catalyst exhibited isolated single-metal structures with minimal interactions between Cu SAs and Ti atoms. In situ DRIFTS analysis confirmed the generation of the intermediate species (*COOH, *CO, *CHO, and *CHOCO) on Cu–Ti–V_O_/Ti_0.91_O_2_–SL catalyst (Fig. 12d). DFT calculations showed that the pristine Ti_0.91_O_2_–SL *CO exhibited a high CO selectivity due to the facile desorption of *CO rather than undergo subsequent hydrogenation or C–C coupling (Fig. 12e). Moreover, C–C coupling on the Cu–O site in the absence of Vos was challenging because of the high uphill energy changes, with hydrogenation of *CO into CH_4_ being more favorable (Fig. 10f). Figure 10g shows the possible pathway of obtaining C_3_H_8_ product Cu–Ti–V_O_ unit. The initial step involved the transformation of absorbed CO_2_ to *CHO via *COOH and *CO intermediates. Next, *CHO at Cu–Ti–V_O_ unit could react with CO coming from adjacent Ti_0.91_O_2_ units, resulting in the formation of *CHOCO intermediates. The subsequent C_1_–C_2_ coupling (*CH_2_OCO + *CO → *CH_2_OCOCO) was found to be a thermodynamically favorable exothermic process. The DFT results revealed that Cu–Ti–V_O_ units have the ability to stabilize *CHOCO and *CH_2_OCOCO intermediates, leading to a reduction in their energy levels. This stabilization was probably due to the largely alleviation of electron accumulation and relaxation of the intermolecular and intramolecular electrostatic repulsion within Cu–Ti–VO units. This mechanism can potentially enable both C_1_–C_1_ and C_1_–C_2_ coupling processes to become favorable exothermic reactions.

#### Cu-Based Heterojunctions

The construction of heterojunctions is regarded as one of the most effective strategies for enhancing the charge separation and transfer processes, consequently improving overall photocatalytic efficiency [115–118]. Copper-based heterojunctions may involve copper in various forms, such as Cu NPs or CuO, combined with other semiconductor materials, in a way that forms a junction between them. For instance, the introduction of Cu (metal, oxide, and quantum dots) as cocatalyst for heterostructures with various semiconductors (such as g-C_3_N_4_, TiO_2_, ZnV_2_O_4_, etc.) was demonstrated to effectively broaden the photoresponse range and effectively improve the PCO_2_RR activity/selectivity toward C_2+_ compounds [119–126]. It is worth noting that despite metal copper itself does not have photocatalytic activity, it has high electrical conductivity, which may enhance the effectiveness of other photocatalysts when combined with them. For instance, Zhao et al. successfully constructed a metal–semiconductor (m–s) heterojunction of Cu-dispersive protonated g-C_3_N_4_ (PCN) through a thermal reduction process of Cu_2_O/PCN, where the total conversion rate of CO_2_ to CH_3_OH and C_2_H_5_OH reached 25.0 μmol g^−1^ under UV–Vis irradiation for 4 h. This value was 4.18 and 1.84 times higher than those obtained from PCN and Cu_2_O/PCN, respectively [123]. The Cu/PCN heterojunction demonstrated a selectivity of 51.42% for CH_3_OH and 46.14% for C_2_H_5_OH. These outcomes indicated that the heterojunction effectively facilitates the separation of charge carriers and inhibits their recombination, resulting in high yield of C_1_ and C_2_ products. In another study, Zhao et al. reported the preparation of metal–semiconductor Cu/ZnV_2_O_4_ heterojunction for the photocatalytic CO_2_ reduction to CH_3_OH and C_2_H_5_OH [124]. They found that composite Cu^0^-ZnV_2_O_4_ increased the surface area and adjusted the energy band position in a way that matched with the reaction potential toward CH_3_OH and C_2_H_5_OH. The improved photocatalytic activity over Cu/ZnV_2_O_4_ was due to the heterojunction interface’s ability to facilitate rapid transmission and hinder the recombination of the photogenerated charges.

In a study conducted by Yu et al., they investigated the impact of depositing CuO_*x*_ onto BiVO_4_ for the photocatalytic conversion of CO_2_ into hydrocarbons [127]. They synthesized monoclinic BiVO_4_ crystals with a truncated tetragonal bipyramidal shape, allowing for controlled ratios of exposed {010} and {110} facets. Notably, they observed that CuO_*x*_ NPs were selectively deposited onto the {010} facets of the BiVO_4_ crystals. Compared to pure BiVO_4_, the CuO_*x*_/BiVO_4_ catalysts, which maintained a uniform truncated tetragonal bipyramidal morphology, exhibited a higher rate of hydrocarbon fuel formation, including CH_4_, C_2_H_6_, and C_3_H_8_. The improved photocatalytic activity was attributed to the enhanced efficiency of charge carrier separation, facilitated by the presence of a Z-scheme junction at the interface between α-CuO_*x*_ and BiVO_4_. In another study [128], Z-type Cu_2_O-modified BiOI microspheres were synthesized through chemical deposition. The incorporation of Cu_2_O onto the surface of BiOI served to enhance the specific surface area of BiOI, providing more exposed active sites. Additionally, the close interaction between Cu_2_O and BiOI facilitated the efficient separation and migration of photogenerated carriers, as well as the use of sunlight. Compared to pristine BiOI, the Cu_2_O/BiOI heterojunction photocatalyst exhibited superior photocatalytic activity. Notably, it resulted in higher yields of CH_3_OH and C_2_H_5_OH CO_2_ photoreduction, with yields reaching 609.05 and 273.96 μmol g_cat_^−1^, respectively.

**Fig. 13:**
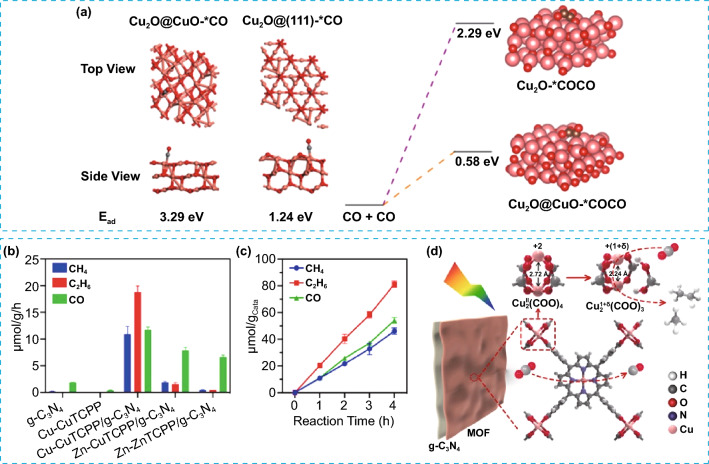
**a** Theoretical calculations of the adsorption energy of *CO and first-principles calculations of the C–C coupling process on Cu_2_O and Cu_2_O@CuO. Reproduced with permission [27]. Copyright 2021, American Chemical Society. **b** PCO_2_RR results on g-C_3_N_4_, Cu-CuTCPP, Cu-CuTCPP/g-C_3_N_4_, Zn-CuTCPP/g-C_3_N_4_, and Zn-ZnTCPP/g-C_3_N_4_, **c** photocatalytic CO_2_ reduction on Cu-CuTCPP/g-C_3_N_4_, and **d** the self-reconstruction of paddle-wheel Cu^II^_2_(COO)_4_ during the PCO_2_RR. Reproduced with permission [125]. Copyright 2022, Elsevier

Besides, Zhao et al. reported the construction of hybrid photocatalyst (CuO_*X*_@p-ZnO) in which CuO_*X*_ is evenly distributed throughout polycrystalline ZnO. This photocatalyst demonstrated the ability to reduce CO_2_ to C_2_H_4_, achieving a production rate of 22.3 μmol g^−1^ h^−1^ and a selectivity of 32.9% [27]. The combination of X-ray absorption fine structure spectra and in situ FT-IR studies demonstrated that Cu was predominantly present as CuO (Cu^2+^) in the initial catalyst. Nevertheless, during the photocatalytic process, a distinctive surface layer of Cu^+^ emerged over the CuO matrix, which served as the active site for capturing in situ generated CO and promoting its transformation into C_2_H_4_ through C–C coupling. In situ FT-IR analysis successfully identified *OC–COH intermediate during the PCO_2_RR, marking the first experimental observation of this intermediate. Additionally, theoretical calculations revealed the significant contribution of Cu^+^ sites in improving the binding of *CO and enhancing the stabilization of the *OC–COH intermediate (Fig. 13a). This study reveals how the Cu valence state could affect the reaction pathway of CO_2_ reduction to produce C_2+_ compounds. The same group recently successfully synthesized a *π*–*π* stacking hybrid structure between g-C_3_N_4_ and 2D MOF of Cu-CuTCPP [125]. The resulting catalyst was able to convert CO_2_ to C_2_H_6_ and achieving a C_2_H_6_ selectivity of 44% (Fig. 13b and c). Interestingly, they identified a light-driving reconstruction of Cu-CuTCPP moiety (Cu^II^_2_(COO)_4_ → Cu^1+δ^_2_(COO)_3_) by the photoinduced electrons from excited g-C_3_N_4_ as depicted in Fig. 13d. The self-reconstruction mainly improved the stabilization of *CO intermediates as well as the synergistic effect of the dual-Cu site, leading to efficient C–C coupling to produce C_2_H_6_.

**Fig. 14:**
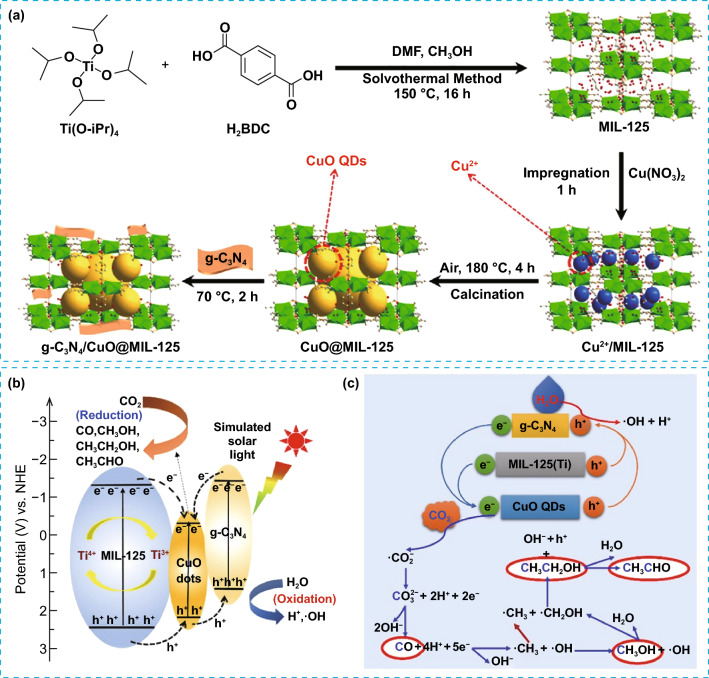
**a** Schematic illustration for the synthesis of g-C_3_N_4_/CuO@MIL-125(Ti) composite photocatalyst, **b** the charge transfer process, and **c** the proposed reaction pathway over g-C_3_N_4_/CuO@MIL- 125(Ti) photocatalyst. Reproduced with permission [126]. Copyright 2020, Elsevier

As mentioned above, the selectivity of Cu-based photocatalysts depends on the oxidation state of Cu. However, the use of Cu in photocatalysis is not yet fully developed as it exhibits poor stability caused by the variations in its oxidation states by the photoinduced charges. Therefore, it is crucial to ensure the stability of Cu to fully utilize its intrinsic photocatalytic properties. To date, the formation of heterojunctions with other photocatalysts is a widely employed approach to improve the stability of Cu. For example, Liu et al. employed a complexation oxidation approach for the encapsulation of CuO QDs in the pores of metal organic framework of MIL-125(Ti) and further combined it with g-C_3_N_4_ to construct a composite photocatalyst, (g-C_3_N_4_/CuO@MIL-125(Ti)), as illustrated in Fig. 14a [126]. This encapsulating structure ensured high stability and reusability of the catalyst. Furthermore, the obtained composite facilitated an efficient electron transfer from MIL-125(Ti) and g-C_3_N_4_ nanosheets to the CuO QDs, boosting the density of electrons over the QDs (Fig. 14b). Consequently, the initial composition of the product shifted from C_1_ (CH_3_OH, CO) to mainly C_2+_ compounds (CH_3_CH_2_OH, CH_3_CHO) for g-C_3_N_4_/CuO@MIL-125(Ti), representing about 77% of the total formed products as shown in Fig. 14c.

In summary, several approaches such as optimising crystal phase and morphology, introducing metal doping, engineering defects, fabricating heterostructures, and utilizing bimetallic synergies have been shown to enhance the efficiency and selectivity of Cu-based materials toward the formation of C_2+_ products in PCO_2_RR. These approaches impact the reaction routes primarily by stabilizing the crucial C_1_ or C_2_ intermediates, decreasing the reaction barrier, offering more active sites, as well as increasing the electron and proton density to facilitate the C–C coupling for the C_2+_ production. Table 2 lists the photocatalytic systems that have been reported for PCO_2_RR into C_2+_ products.

**Table 2:** Summary of activity of Cu-based photocatalysts for C_2+_ products

Photocatalyst	Light source	Main products	Yield	References
Cu_0.33_-Pt_0.67_/TiO_2_	Solar simulator 32 cm. AM1.5 sunlight	CH_4_	2.60 mL g^−1^ h^−1^,	[37]
		C_2_H_4_	0.33 mL g^−1^ h^−1^	
		C_2_H_6_	0.47 mL g^−1^ h^−1^	
Three-dimensional porous Cu_2_O	300 W Xe lamp (λ > 420 nm)	CO	26.8 μmol g^−1^ h^−1^	[84]
		CH_4_	4.04 μmol g^−1^ h^−1^	
		C_2_H_4_	0.66 μmol g^−1^ h^−1^	
CdS/(Cu-TNTs)	450 W Xe lamp (λ > 420 nm)	CH_4_	49.1%	[86]
		C_2_H_4_	1.3%	
		C_2_H_6_	31.3%	
		C_3_H_6_	0.5%	
		C_3_H_8_	17.9%	
AuCu/g-C_3_N_4_	300 W Xe lamp (λ > 420 nm)	CH_3_OH	0.14 mmol g^−1^ h^−1^	[87]
		CH_3_CH_2_OH	0.89 mmol g^−1^ h^−1^	
Cu_0.8_Au_0.2_/TiO_2_	300 W xenon lamp (light intensity, 500 mW cm^−2^)	CH_4_	3578.9 μmol g^−1^ h^−1^	[88]
		C_2_H_4_	369.8 μmol g^−1^ h^−1^	
Cu–Pt/BT (blue titania)	Xe-arc lamp with an AM 1.5 filter (100 W, 100 mW cm^−2^)	CH_4_	3.0 mmol g^−1^ h^−1^	[89]
		C_2_H_6_	0.15 mmol g^−1^ h^−1^	
AgCu-TNTA	Solar simulator, AM1.5 sunlight	CH_4_	9.38 μmol g^−1^ h^−1^	[15]
		C_2_H_6_	14.5 μmol g^−1^ h^−1^	
CuGaS_2_/Ga_2_S_3_	300 W Xe lamp with a 420 nm cutoff filter (*λ* > 420 nm)	CO	51 μmol g^−1^ h^−1^	[93]
		CH_4_	19 μmol g^−1^ h^−1^	
		C_2_H_4_	335 μmol g^−1^ h^−1^	
CuACs/PCN	300 W Xe lamp (λ > 420 nm)	CH_4_	26.85 μmol g^−1^	[100]
		C_2_H_4_	30.51 μmol g^−1^	
Cu SAs/UiO-66-NH_2_	300 W Xe lamp with a cut-off filter of 400 nm	CH_3_OH	5.33 μmol g^−1^ h^−1^	[101]
		CH_3_CH_2_OH	4.22 μmol g^−1^ h^−1^	
Re-bpy/PTF(Cu)	300 W Xe lamp (λ > 420 nm)	CO	-	[102]
		CH_4_	-	
		C_2_H_4_	73.2 μmol g^−1^ h^−1^	
0.02%Co-1%Cu/TiO_2_	Solar simulator, AM1.5 sunlight	CO	135.94 μmol	[103]
		CH_4_	127.05 μmol	
		C_2_H_6_	267.60 μmol	
		C_3_H_8_	10.07 μmol	
InCu/PCN	Solar simulator, AM1.5 sunlight	CO	5.8 μmol g^−1^ h^−1^	[104]
		CH_4_	1.2 μmol g^−1^ h^−1^	
		CH_3_CH_2_OH	28.5 μmol g^−1^ h^−1^	
Cu^δ+^/CeO_2_-TiO_2_	300 W Xe lamp (light intensity 200 mW cm^−2^)	CO	3.47 μmol g^−1^ h^−1^	[105]
		CH_4_	1.52 μmol g^−1^ h^−1^	
		C_2_H_4_	4.51 μmol g^−1^ h^−1^	
CuPt/WO_3_	300 W xenon lamp	HCOOH	2.62 μmol g^−1^ h^−1^	[106]
		CH_3_COOH	19.41 μmol g^−1^ h^−1^	
Cu-Ti-V_O_/Ti_0.91_O_2_-SL	300 W Xe lamp	CO	18.6 μmol g^−1^ h^−1^	[107]
		C_2_H_4_	7.6 μmol g^−1^ h^−1^	
		C_3_H_8_	13.8 μmol g^−1^ h^−1^	
15 wt% Cu/GO	Halogen lamp (light intensity 100 mW cm^−2^)	CH_3_CHO	1.79 μmol g^−1^ h^−1^	[119]
SCN-Cu/TiO_2_-SBO Defective titania	300 W xenon lamp simulated sunlight	CO	2.3 µmol g^−1^ h^−1^	[121]
		CH_4_	10 µmol g^−1^ h^−1^	
		C_2_H_4_	4.8 µmol g^−1^ h^−1^	
Nb_2_O_5_/CuO 2.5%	UVC lamp (light intensity 21.49 mW cm^−2^)	CH_4_	28 µmol g^−1^	[122]
		HCOOH	7.5 µmol g^−1^	
		CH_3_COOH	72 µmol g^−1^	
Cu/PCN	300 W xenon lamp (luminous power of 2.5 W)	CH_3_OH	13.8 µmol g^−1^	[123]
		CH_3_CH_2_OH	11.2 µmol g^−1^	
Cu/ZnV_2_O_4_	300 W xenon lamp	CO	–	[124]
		CH_4_	–	
		CH_3_OH	3.30 µmol g^−1^ h^−1^	
		CH_3_CH_2_OH	0.86 µmol g^−1^ h^−1^	
CuO_*X*_@p-ZnO	300 W xenon lamp (150 mW cm^−2^)	CO	27.3 µmol g^−1^ h^−1^	[27]
		CH_4_	17.9 µmol g^−1^ h^−1^	
		C_2_H_4_	22.3 µmol g^−1^ h^−1^	
Cu-CuTCPP/g-C_3_N_4_	300 W xenon lamp with both UV-cut and IR-cut filter (150 mW cm^−2^)	CO	12.3 µmol g^−1^ h^−1^	[125]
		CH_4_	11.6 µmol g^−1^ h^−1^	
		C_2_H_6_	18.5 µmol g^−1^ h^−1^	
g-C_3_N_4_/CuO@MIL-125(Ti)	300 W xenon lamp	CO	180.1 µmol g^−1^	[126]
		CH_3_OH	997.2 µmol g^−1^	
		CH_3_CH_2_OH	1505.7 µmol g^−1^	
		CH_3_CHO	531.5 µmol g^−1^	

### Electrocatalytic CO_2_ Reduction Reaction

The selection of the right electrocatalyst is a critical factor that determines the efficacy ECO_2_RR process. It impacts the reaction kinetics, selectivity of possible products, and the required overpotential. So far, copper-based materials are the most effective electrocatalysts for ECO_2_RR to C_2+_ products, but they are still relatively unselective. This is owing to the moderate binding affinity of copper to carbon monoxide which allows the generation of a wide range of products including, but not limited to, methane, methanol, ethylene, ethane, ethanol, and propanol [129–136]. Nevertheless, the origin of the enhanced activity/selectivity of Cu-based materials in ECO_2_RR toward C_2+_ chemicals production is not easily identified. To date, many factors have been shown to affect the overall mechanism to C_2+_ products including the experimental setup conditions (pH, cation/anion of electrolyte, temperature, pressure, and applied overpotential) [137–143] and the catalyst surface properties (morphology, oxidation states, exposed facets, and defects) [144–148]. In this section, the relationship between the Cu surface and the performance of ECO_2_RR is discussed, with the aim of comprehending the origin of the improvement observed in C_2+_ production over Cu-based materials. The discussion is approached from a materials viewpoint, and the Cu-based materials for ECO_2_RR are categorized into three groups: Cu metal/oxides, Cu alloys, and Cu-based single-atom catalysts (Cu SACs). Table 3 provides a summary of the use of Cu-based catalysts in ECO_2_RR to C_2+_ compounds.

**Table 3:** Summary of recent Cu-based catalysts applied for CO_2_RR to C_2+_ products

Catalyst	Electrolyte	Potential	Products	FE (%)	References
Cu/CNS	0.1 M KHCO_3_	− 1.2 V_RHE_	CH_3_CH_2_OH	63	[134]
Cu_2_O	0.1 M KHCO_3_	− 0.99 V_RHE_	C_2_H_4_	39	[136]
			CH_3_CH_2_OH	16	
Cu cubes	0.1 M KHCO_3_	− 1.1 V_RHE_	C_2_H_4_	41	[153]
Cu foils	0.1 M KHCO_3_	− 0.9 V_RHE_	C_2_H_4_	60	[155]
Cu_2_O	0.1 M KHCO_3_	− 1.1 V_RHE_	C_2_H_4_	57.3	[156]
Cu	0.1 M KHCO_3_	− 1.2 V_RHE_	C_2_H_4_ + CH_3_CH_2_OH + CH_3_COOH	54	[150]
Gd/CuO_*x*_	2 M KOH	− 0.8 V_RHE_	C_2_H_4_ + CH_3_CH_2_OH + CH_3_COOH + n-Pr	81.4	[164]
Zn-Cu	0.1 M KHCO_3_	− 1.1 V_RHE_	C_2_H_4_	33	[168]
Ag–Cu	0.1 M KHCO_3_	− 1.1 V_RHE_	C_2_H_4_	37	[169]
Cu-Ag	1M KOH	− 0.7 V_RHE_	C_2_H_4_	60	[173]
			CH_3_CH_2_OH	25	
Ag_*x*_Cu_100−*x*_ Cu rich	0.5 M KHCO_3_	− 0.9 V_RHE_	C_2_H_4_	60.3	[174]
			CH_3_CH_2_OH		
HKUST-1	1M KOH	− 1.07V_RHE_	C_2_H_4_	45	[179]
Cu(OH)BTA	1M KOH	− 0.87 V_RHE_	C_2_H_4_	57	[182]
			CH_3_CH_2_OH	11	
			CH_3_COOH	4	
			n-Pr	1	
Cu-SA/NPC	0.1 M KHCO_3_	− 0.76 V_RHE_	CH_3_COCH_3_	36.7	[183]

#### Cu Metal/Oxides

The copper surface morphology and geometry have significant effects on the type of products generated during ECO_2_RR. The faradic efficiencies (FE) for the formation of methane and C_2_ products (ethylene and ethanol) on polycrystalline Cu surfaces at − 5 mA cm^−2^ in 0.1 M KHCO_3_ are approximately 29% and 37%, respectively. The presence of heterogeneous catalytic sites on the polycrystalline Cu plane may account for the insufficient selectivity [135]. Significant improvements were found by using single-crystal Cu(100) and cleaved Cu(100) surfaces with high-indexed planes. For example, Cu(S)-[4(100) × (111)] surface showed the formation of ethanol and ethylene with a total FE of about 57% [149]. It is suggested that the atomic steps and a square arrangement of Cu atoms in the (100) terraces facilitate the coupling of CH_*x*_O intermediates, thereby contributing to the generation of more C_2+_ products. Even though Cu(100) single crystals are the optimal for ethylene production, they still produce a considerable quantity of methane. However, the use of Cu cubes with (100) facets, having an edge length of approximately 100 nm, can further enhance ethylene selectivity by almost completely suppressing methane formation [150].

**Fig. 15:**
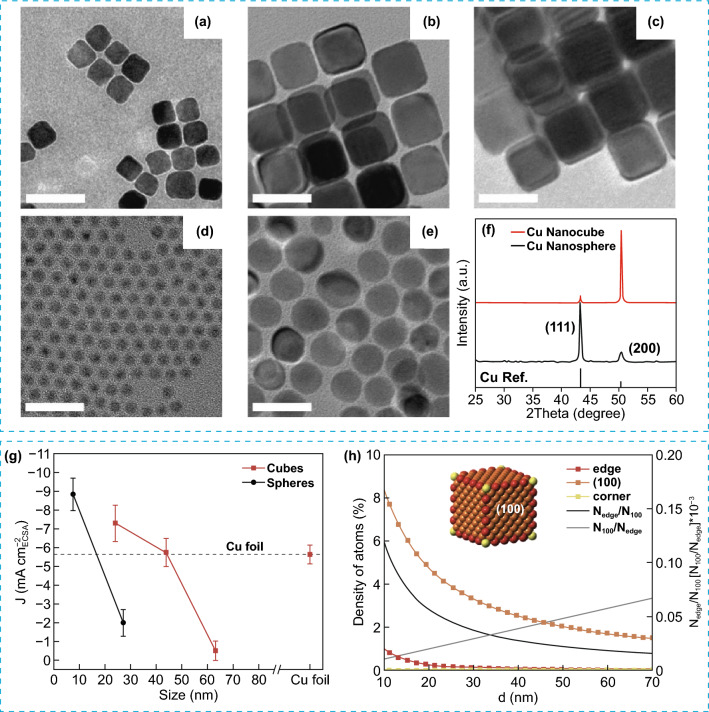
TEM images of Cu cubes with an average edge length of **a** 24 nm, **b** 44 nm, **c** 63 nm, TEM images of Cu spheres with an average diameter of **d** 7.5 nm, **e** 27 nm, **f** XRD patterns of the Cu cubes and Cu spheres, **g** current density at − 1.1 V_RHE_ plotted versus the size of the Cu NC catalysts, and **h** density of adsorption sites in Cu NC cubes (left axis) and trend Nedge/N100 and N100/Nedge (right axis) versus the edge length. Reproduced with permission [151]. Copyright 2016, Wiley–VCH

In a study by Buonsanti et al., the influence of size and shape of Cu nanocrystals (NCs) on the activity and selectivity in ECO_2_RR was investigated [151]. By using colloidal chemistry approach, Cu NC spheres with two distinct sizes (7.5 and 27 nm) and Cu NC cubes with three different sizes (24, 44, and 63 nm) were synthesised (Fig. 15a–e). The X-ray diffraction patterns for Cu NC cubes and spheres were compared, revealing that the Cu cubes were dominated by {100} facets, as indicated by the more pronounced (200) peak compared to the bulk fcc Cu reference (Fig. 15f). The results showed that, while smaller nanoparticles with the same morphology demonstrated greater activity, the cube-shaped nanoparticles displayed greater intrinsic activity compared to the spheres (Fig. 15g). A noteworthy observation was the nonlinear trend in selectivity, as Cu cube nanoparticles with a side length of 44 nm exhibited 80% selectivity toward carbon products, of which 50% was identified as ethylene. The superior activity observed in the Cu NC cubes (44 nm) can be attributed to the optimal proportion of edge sites to (100) plane sites (*N*_edge_/*N*_100_ = 0.025), as evidenced by the statistical analysis of the surface atom density (Fig. 13h), which emphasizes the crucial role of edge atoms in the active sites that selectively drive ECO_2_RR and ethylene production in these Cu nanocrystal cubes.

**Fig. 16:**
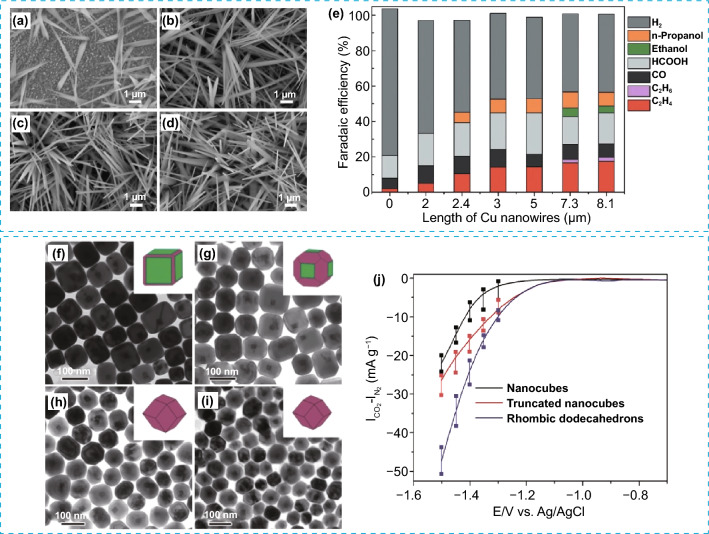
**a–d** SEM images of Cu(OH)_2_ nanowires with synthesis time of 1, 3, 5, and 8 min, respectively, **e** FE for different products on Cu nanowire arrays with different lengths at − 1.1 V_RHE_ in CO_2_-saturated 0.1 M KHCO_3_ electrolytes. Reproduced with permission [152]. Copyright 2016, Wiley–VCH. **f-i** TEM images of as-etched Cu NCs subjected to different etching periods of 4 h, 8 h, 12 h, and 24 h, respectively. Reproduced with permission [153]. Copyright 2016, American Chemical Society

In another study, researchers explored the effect of copper nanowire (Cu NW) morphology on ECO_2_RR toward C_2+_ hydrocarbons [152]. It was observed that the selectivity for C_2+_ hydrocarbons, on Cu NWs array electrodes could be finely adjusted by systematically modifying the Cu NW length and density, which could be controlled by varying the reaction times (Fig. 16a–d). Their findings indicated a gradual increase in the formation of C_2_H_4_ as the length and thickness of Cu nanowires increased. As shown in Fig. 15e, at a length of 8.1 mm for the Cu NWs, the FE for C_2_H_4_ formation reached 17.4%, while the production of H_2_ is simultaneously reduced.

Nanomaterials with controlled morphology play a pivotal role in both assessing the effect of different facets on the ECO_2_RR and designing catalysts with superior performance and selectivity. In this regard, Yin and colleagues [153] reported the synthesis of cubic Cu NPs through chemical etching, spanning across different time intervals, leading to the formation of various shapes as illustrated in Fig. 16f–i. Notably, when the nanocrystals were etched for a duration exceeding 12 h, they exhibited a rhombic dodecahedral morphology, prominently exposing high-energy (110) facets. When compared to Cu nanocubes, rhombic dodecahedral Cu NPs exhibit a positive onset potential of − 1.1 V_RHE_. Additionally, at − 1.4 V_RHE_, the current density for the rhombic dodecahedral structure is approximately three times higher than that of Cu nanocubes. This observation indicates that the Cu crystal structure with enriched (110) facets outperforms the one with (100) facets in terms of catalytic activity as depicted in Fig. 16j. The presence of high-energy (110) facets in the rhombic dodecahedral Cu structure leads to increased selectivity for the formation of C_2+_ products, including C_2_H_4_, C_2_H_6_, and C_3_H_8_ when compared to the original (100) facet of cubic Cu. These findings suggest that the (110) facet of rhombic dodecahedral Cu is particularly conducive to the formation of C_2+_ hydrocarbons in ECO_2_RR.

In addition to considering structural parameters including particle size/shape and reactive facets, it has been suggested that the surface state of Cu is an important factor influencing the activity and product selectivity in ECO_2_RR. Recently, it was demonstrated that the interaction between the surfaces made of Cu^+^ and Cu^0^ restrains the C_1_ pathways while promoting CO–CO dimerization owing to the attraction between carbon atoms with opposite charges stimulated by the interface between Cu^+^ and Cu^0^ [154]. According to a recent study, oxide-derived copper catalysts, activated using oxygen plasma, showed a noteworthy ethylene FE of 60% at − 0.9 V_RHE_. This high efficiency was related to the existence of Cu^+^ species. Interestingly, through the in situ hard X-ray absorption spectroscopy (hXAS) analysis, it was observed that stable Cu^+^ species were detected at notably negative potentials of about − 1.0 V_RHE_ during the ECO_2_RR [155].

In a study conducted by Hwang et al., it was found that the initial stages of ECO_2_RR caused fragmentation of Cu_2_O NPs, leading to improved C_2_H_4_ selectivity and activity [156]. The researchers utilized a cysteamine immobilization agent to synthesize Cu_2_O nanoparticles by direct growth on carbon black, which initially measured at a size of 20 nm, but were found to be broken down into smaller sizes (2–4 nm) with denser arrangement and deformed crystal planes after 10 h of ECO_2_RR. These morphological changes over reaction time led to higher activity, with a C_2_H_4_ FE of 57.3% achieved at − 1.1 V_RHE_ in 0.1 M KHCO_3_. In situ/operando XANES analysis revealed a shift in oxidation state from Cu^+^ to Cu^0^, suggesting that Cu^0^ was the dominant state during the electrochemical CO_2_ reduction, even in Cu catalysts derived from the oxide material. Upon fragmentation of the initial Cu NPs, they underwent re-oxidation to Cu^+^ states without air exposure, whereas large Cu NPs retained their Cu^0^ state when subjected to open-circuit potential conditions, suggesting that the morphological alterations observed in fragmented Cu_2_O nanoparticles enable greater oxygen access and contribute to improved CO_2_ reduction efficiency.

In another study, Sargent et al. reported the electro-redeposition of Cu (ERD Cu) from a Cu_2_(OH)_3_Cl sol–gel precursor to control the morphology and oxidation state of Cu in order to inhibit CH_4_ formation and enhance C_2_H_4_ production [150]. Various structural morphologies appeared as a function of applied potential, including rounded nanostructures (at − 0.7 V_RHE_), sharper needles (at − 1.0 V_RHE_), sharper nanowhiskers (at − 1.2 V_RHE_), and dendrites with rounded tips (at − 1.4 V_RHE_). The ERD Cu catalyst was found to exhibit a 54% FE for C_2+_ products (ethylene, acetate, and ethanol) and an 18% FE for C_1_ products (carbon monoxide, methane, and formate) at − 1.2 V_RHE_. Additionally, the C_2_H_4_ partial current density was high for ERD Cu in both the H-cell (22 mA cm^−2^ at − 1.2 V_RHE_) and flow cell (161 mA cm^−2^ at − 1.0 V_RHE_) configurations accompanied by notable inhibition of CH_4_ with a C_2_H_4_/CH_4_ ratio of 200. Notably, the high local pH induced by ERD Cu is an important factor in the suppression of CH_4_. This is because ERD Cu generated remarkably high current densities, with a recorded 60 mA cm^−2^ in H-cell and 450 mA cm^−2^ in flow cell, resulting in a rapid consumption of protons that led to the elevated local pH [157, 158]. At high pH levels, CO dimerization pathway is most likely to occur because the CO hydrogenation step is kinetically limited. At very negative potentials (less than − 1.0 V_RHE_), in situ soft X-ray absorption (sXAS) spectroscopy indicated the existence of Cu^+^ species. For over 1 h of operation, it was up to 23% of the ERD Cu electrocatalyst consisted of Cu^+^ species, even when subjected to a negative applied bias as low as − 1.2 V_RHE_, indicating a crucial role of Cu_2_(OH)_3_Cl sol–gel in stabilizing Cu^+^ species at higher negative applied potentials. According to DFT calculation, it was observed that the ERD Cu(211) model, comprising of 25% Cu^+^ species, closely resembling the optimal Cu^+^ quantity identified through sXAS analysis, demonstrated the minimum Gibbs free energy of formation of the OCCOH* intermediate (1.13 eV). This value was 0.76 eV lower than that of the Cu(211) model. Moreover, the ERD Cu(211) surface showed the highest CO binding, as evidenced by a binding energy of − 1.45 eV, indicating that the Cu^+^ species has a significant impact on the stabilization of the OCCOH* intermediate, thereby promoting the formation of C_2_ products over C_1_ products. Overall, the Cu^+^ site plays a crucial role in enhancing the selectivity toward C_2+_ products by promoting the C–C coupling reaction through strengthened *CO adsorption and stabilized C_2_ intermediates. Nevertheless, the low stability of Cu^+^ species at negative potentials during the ECO_2_RR remains a major impediment to its large-scale implementation. To address this challenge, metal doping is suggested as effective way to boost the stability of Cu^+^ species [159–163].

Recently, the study conducted by Han et al. applied DFT calculations and multiple characterization tests to investigate the impact of atomic dopants on the efficiency of Cu-based catalysts or CO_2_ electroreduction to C_2+_ products [164]. Starting with DFT calculations, they explored the effectiveness of doping various single-atom metals (Ag, Pd, Ni, Zn, Sn, Ce, Sm, and Gd) of Cu_2_O and identified single-atom Gd-doped Cu_2_O as a highly promising choice for ECO_2_RR to C_2+_ products. This was attributed to its ability to enhance the CO_2_ conversion to *CO, suppress HER, demonstrated lower thermodynamic limiting potentials for ECO_2_RR, and exhibited moderate *CO binding energy. The characterization results of as-prepared Gd/CuO_*x*_ confirmed that has the ability to stabilize Cu^+^ species and induce tensile strain in the catalyst. The Gd/CuO_*x*_ catalyst exhibited superior CO_2_ conversion to C_2+_ product, with a Faradaic efficiency of 81.4% and a partial current density of C_2+_ product reaching 444.3 mA cm^−2^ at − 0.8 V_RHE_. Both DFT calculations and experiments revealed that the stability of the key intermediate O*CCO was enhanced by Gd doping. Additionally, the energy barrier for the CO dimerization to O*CCO step is significantly affected by tensile strain, ultimately contributing to the superior performance of the Gd/CuO_*x*_ catalyst in ECO_2_RR to C_2+_ products.

#### Cu Alloys

**Fig. 17:**
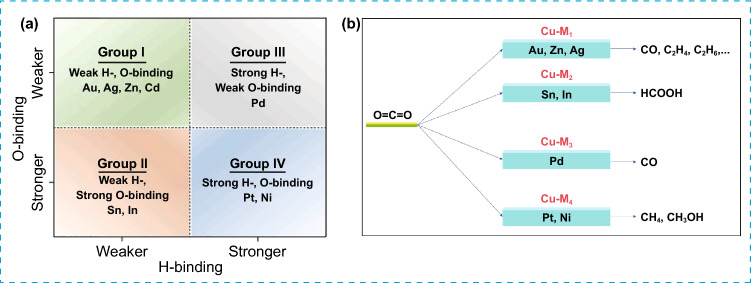
**a** Classification of various metals into four groups in relation to Cu alloys based on O and H affinities from the previous studies. Reproduced with permission [165]. Copyright 2018, Elsevier. **b** Reaction products of electrocatalytic CO_2_ reduction on different Cu alloys

The introduction of a secondary metal to single Cu crystal can alter its chemical composition, leading to improved activity/selectivity by adjusting the binding strength of key intermediates on the catalyst surface. Thus, Cu alloys have garnered significant attention in this regard. Based on the previous experiments, Qiao et al. [165] introduced a classification system for the secondary metal of Cu alloys based on their relative affinities toward H and oxygen O, as illustrated in Fig. 17a. They proceeded to examine the distinct catalytic mechanisms and resultant product variations associated with each of these four affinity categories separately. For ECO_2_RR, metals that exhibit a higher affinity for O but a lower affinity for H compared to Cu, such as Sn, In, Hg, and Pb, tend to result in the formation of a weakly bound *COOH intermediate after the initial reaction step. Consequently, the primary product of CO_2_ reduction on these metal surfaces is HCOOH. There is even evidence to suggest that the reaction may proceed through an O-bound *OCHO intermediate on these metals. Conversely, metals with lower affinities for both O and H than Cu, including Zn, Ag, and Au, exhibit a stronger binding affinity for *COOH compared to *CO. Consequently, CO is desorbed as the primary product, with the formation of *COOH serving as the potential determining step [166]. On the other hand, metals that possess both a stronger affinity for O and H than Cu, which include Co, Ni, Fe, and Pt, generally tend to favor the competitive HER with a minor amounts of hydrocarbons and alcohols being detected on the surfaces of these metal catalysts.

It was found that when group 1 metals including Au, Zn, and Ag (denoted as M_1_) are the predominant component in Cu–M_1_ systems, the primary product is CO, and the FE is generally better than that of the parent metals. This outcome is likely due to the increased O affinity of Cu, which stabilizes the *COOH intermediate, while the weak binding ability of M_1_ metals for *CO facilitates the desorption of CO. However, in cases where M_1_ provides a moderate contribution to the active sites, C_2+_ products are generally produced with a high FE due to the synergistic effect between Cu and M_1_. Moreover, the weak binding of H also suppresses HER, enhancing the overall selectivity for ECO_2_RR (as illustrated in Fig. 17b). For group 2 metals (M_2_), the trend suggests a preference for 2e^−^ reduction products in Cu-M_2_ systems, likely due to the weak H binding, which inhibits pathways beyond CO. When M_2_ metals dominate the active sites, HCOOH becomes the major reduction product. This is attributed to the increased availability of O-binding sites, potentially leading to better stabilization of the *OCHO intermediate and a favorable pathway to HCOOH formation. It is noteworthy that there is limited research on the coupling of group 3 metals (M_3_, specifically Pd) with Cu, and the intermetallic arrangement of these materials significantly influences product selectivity. When the metallic arrangement is more regular, CO becomes the major product and is produced with a high FE. There are fewer examples of group 4 metals including Pt and Ni (M_4_) being coupled with Cu in the literature. Although M_4_ metals with a strong affinity for CO should be avoided, these materials can produce CH_4_ and CH_3_OH with relatively high FE, indicating their potential for specific applications in ECO_2_RR [165].

As mentioned above, the synergetic effect between Cu and group 1 metals (e.g., Au, Ag, or Zn) has been reported to improve the production of C_2+_ compounds [160, 167–171]. Specifically, the *CO dimerization process (which generates *OCCO) on bimetallic Cu surfaces could be facilitated owing to the enhanced reduction of CO_2_ to CO via Au, Ag, or Zn sites. For instance, Kuhl et al. reported that the selectivity toward C_2+_ products of Au–Cu alloys was significantly higher than that of a monometallic Cu catalyst at − 0.7 V_RHE_ using 0.1 M KHCO_3_ as electrolyte [160]. In a study by Du et al., it was found that of C_2_H_4_ FE increased from 15 to 33% for a Cu and a Cu_4_Zn_1_ alloy catalyst, respectively [168]. Meanwhile, Buonsanti et al. explored Ag–Cu alloys and determined that, when operating at − 1.1 V_RHE_, using 0.1 M KHCO_3_ as electrolyte, the C_2_H_4_ FE increased from 13 to 37% with Ag_1_–Cu_1.1_ alloy catalyst, as compared to the pure Cu catalyst [169]. Based on these studies, the guest metals (Au, Ag, and Zn) can boost the CO concentration near the catalyst surface, making it easier for Cu sites to convert it into C_2+_ products. The disposition of the guest metals on the surface of copper has the potential to alter the electronic states of the catalyst and to modify the interatomic distance between surface atoms and influence the reaction kinetics of the adsorbed intermediates on the surface. These factors may ultimately affect the chemical binding strengths of reaction, thereby modulating the reaction toward pathways that are more advantageous [171, 172]. For example, recent experiments by Timoshenko et al. using in situ EXAFS demonstrated how the composition and structure affect the product distribution of CO_2_RR. The researchers observed that Cu-ZnNPs with shorter interatomic distances have a greater affinity for producing CH_4_, whereas NPs with longer Cu–Zn distances are more likely to generate CO, which is then reduced to C_2+_ compounds [172].

**Fig. 18:**
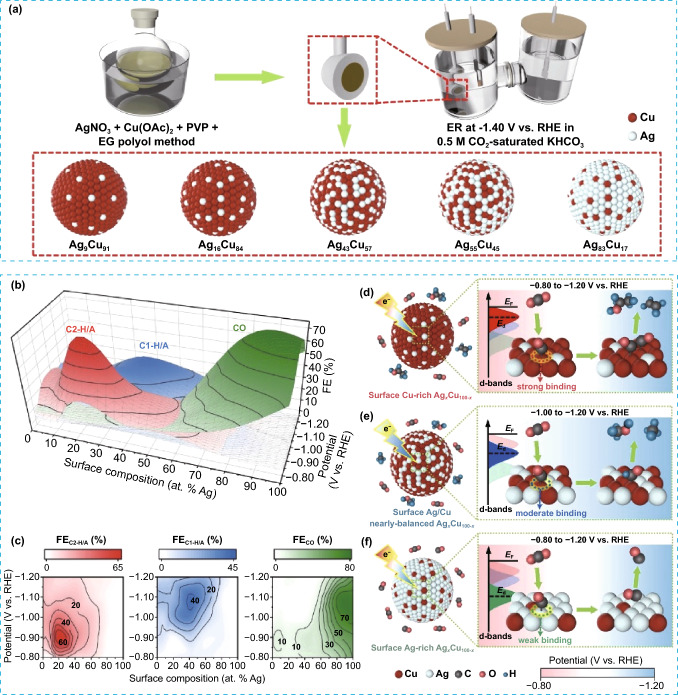
**a** Schematic illustration of the synthetic processes of Ag_*x*_Cu_100−*x*_ alloys, **b** 3D colormap surface plot, **c** corresponding colortour maps for FE of different products vs. surface compositions and applied cathodic potentials, mechanism illustrations of selective production on Ag_*x*_Cu_100−*x*_ alloys, **d** C2H/A over surface Cu-rich, **e** C1H/A over surface Ag/Cu nearly-balanced, and **f** CO over surface Ag-rich. Reproduced with permission [174]. Copyright 2023, Wiley–VCH

Related to the high levels of C_2+_ selectivity at high activity challenges (current density << − 200 mA cm^−2^, applied potentials >> − 1.0 V_RHE_) on Cu alloy-based catalysts, Gewirth et al. demonstrated the enhanced selectivity of ECO_2_RR toward ethylene and ethanol with bimetallic CuAg catalyst synthesized by using additive-controlled electrodeposition method [173]. In an alkaline flow electrolyzer, the CuAg catalyst that has a nanoporous structure and a small amount of Ag (< 10%) showed high ethylene selectivity (~ 60%) and ethanol production (~ 25%) at a relatively low applied potential of − 0.7 V_RHE_ and a high current density of − 300 mA cm^−2^. According to the findings from in situ Raman analysis and control experiments, it can be inferred that the improved selectivity toward ethylene and ethanol can be ascribed to the presence of an optimal amount of Ag responsible for stabilizing the Cu_2_O overlayer and to the higher flux of CO generated by the active promoter Ag that generated more CO intermediates during the ECO_2_RR process. Recently, Shen et al. reported the use of Ag–Cu bimetallic surface alloys (Ag_*x*_Cu_100−*x*_) prepared by a modified polyol method followed by electrochemical reduction as illustrated in Fig. 18a [174]. The chemical compositions of Ag_*x*_Cu_100−*x*_ alloys were rationally adjusted from Cu-rich to Ag-rich. Interestingly, different products including CO, C1 hydrocarbons/alcohols (C1-H/A) and C2 hydrocarbons/alcohols were selectively obtained over Ag_*x*_Cu_100−*x*_ alloys by modulating the surface chemical compositions and applied potentials. The findings indicated that the Ag_*x*_Cu_100−*x*_ alloys catalysts, having Cu-rich surface ranging from 14 to 34 at%, exhibited good selectivity toward C2-H/A products (C_2_H_4_ and C_2_H_5_OH) when subjected to applied potentials within the range of − 0.85 to − 1.0 V_RHE_, as illustrated in Fig. 18b and c. For example, Ag_16_Cu_84_ catalyst showed a high FE_C2-H/A_ of 60.3% at − 0.90 V_RHE_. Meanwhile, the increase in surface Ag compositions (Ag: 27–55 at%) in AgxCu_100−*x*_ alloys resulted in an enhanced formation of C1-H/A (namely, CH_4_ and CH_3_OH) with applied potentials shifted from − 1.00 to − 1.10 V_RHE_. Specifically, the nearly-balanced Ag/Cu surface Ag_43_Cu_57_ displayed a high FE_C1-H/A_ reaching 41.4% at − 1.10 V_RHE_. As expected, the Ag-rich surface alloys (Ag: > 74 at%) selectively convert CO_2_ to CO at applied potentials between − 0.90 and − 1.15 V_RHE_. As example, the Ag_83_Cu_17_ catalyst exhibited a high FE_CO_ of 74.0% at − 1.10 V_RHE_. The findings from DFT calculations show that the high-energy levels of *d*-band centers (E_*d*_) on the surface of Cu-rich Ag_*x*_Cu_100−*x*_ result in a strong binding affinity for *CO and allow for coupling of *CO into *COCO over a wide potential range (− 0.80 to − 1.20 V_RHE_), leading to a yield of C2H/A with high selectivity. For Ag/Cu balanced Ag_*x*_Cu_100−*x*_ alloys (Fig. 18e), a moderate binding strength of *CO was determined by the downshifted E_d_ (− 3.24 to − 3.80 eV), which renders the hydrogenation of *CO to *CHO as predominate reaction, leading to selective formation of C1H/A products at high potential (more negative than − 1.0 V_RHE_). However, in the Ag-rich Ag_*x*_Cu_100−*x*_ alloys (Ag: > 60 at%), the further decreased E_d_ (< − 4.08 eV) resulting in weaker binding of *CO intermediates on the surface (Fig. 18f), which facilitate the desorption of *CO intermediates from the surface to generate CO with high selectivity across a wide range of potentials (− 0.80 to − 1.20 V_RHE_). This study presents a potential avenue for controlling the selectivity of bimetallic alloys to produce desired value-added chemicals.

#### Cu SACs

Catalysts consisting of a single atom (SAs) with active metal centers distributed at the atomic level have been found to exhibit improved activity and tunable selectivity in ECO_2_RR. This can be attributed mainly to the outstanding effectiveness in atom utilization, distinctive electronic structure, and unsaturated coordination surroundings of the metallic centers [175–177]. Considering that the CO_2_RR occurs over solid–liquid–gas boundaries, maximizing the surface area is crucial in order to attain optimal electron and proton transfer across these interfaces. This can be achieved by reducing the CO_2_RR to a single-atom scale and utilizing substrates that are highly porous and stable. Cu-SACs are of great interest among the SACs due to their remarkable ability to produce high C_2+_ products with high selectivity. N-doped conductive carbon supports with N-chelated metal sites are frequently used examples for ECO_2_RR. For instance, Guan et al. reported the use of a nitrogen coordination strategy for dispersing SAs Cu catalysts on nitrogen-doped carbon [178]. The incorporation of nitrogen promoted the dispersion and interaction of atomic copper species onto carbon frameworks doped with nitrogen via Cu–N_*x*_ coordinations. The study revealed that when the Cu concentration reached 4.9%mol, the close proximity of Cu–N_*x*_ species was sufficient to facilitate C–C coupling, leading to the generation of C_2_H_4_. Conversely, when the Cu concentration was below 2.4%mol, the gap between Cu–N_*x*_ species was significant, favoring the production of CH_4_. According to DFT calculations, the generation of C_2_H_4_ was facilitated by coupling two *CO intermediates on adjacent Cu–N_2_ sites, whereas isolated Cu–N_2_ and Cu–N_4_ sites as well as neighboring Cu–N_4_ sites promoted the production of CH_4_. Despite the promising performance of Cu-SACs, there is still a need for more improvement in terms of C_2+_ product selectivity and Faradaic efficiency. This is mainly due to the existence of ambiguous catalytic sites and the adverse electronic properties of Cu when coordinated with N.

**Fig. 19:**
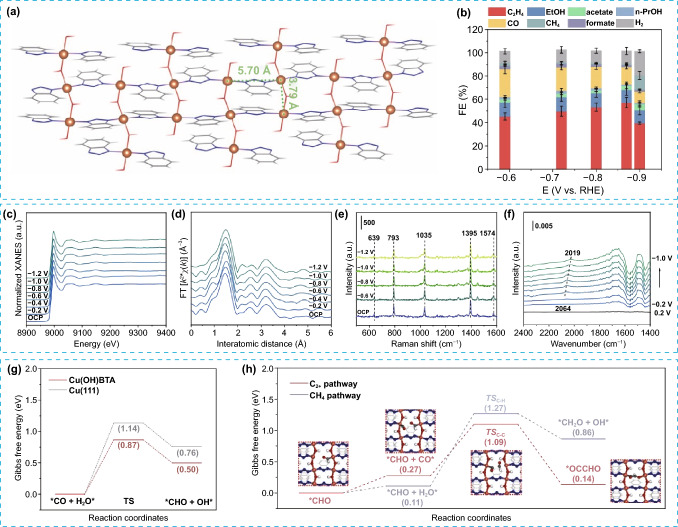
**a** The proposed model of Cu(OH)BTA with isolated Cu atoms, **b** FEs of all products at different applied potentials over Cu(OH)BTA, **c** operando Cu K-edge XANES, **d** EXAFS of Cu(OH)BTA under CO_2_ atmosphere at applied potentials ranging from − 0.2 to − 1.2 V, **e** in situ Raman spectra of Cu(OH)BTA under CO_2_ atmosphere at the applied potential of − 0.6 to − 1.2 V, **f** in situ ATR-SEIRAS spectra of Cu(OH)BTA in CO_2_-saturated 0.1 M KHCO_3_ electrolyte at the applied potential of − 0.2 to − 1.0 V, **g** Gibbs free energy diagram of *CO hydrogenation on Cu(OH)BTA slab and Cu(111) slab, and **h** Gibbs free energy diagram of ECO_2_RR to C_2+_ and CH_4_ pathway on Cu(OH)BTA slab. Reproduced with permission [182]. Copyright 2023, Nature

According to reports, Cu-SACs have the ability to facilitate the crucial C–C coupling reaction, which is necessary for C_2+_ products formation. Nonetheless, it was proven that these catalysts undergo in situ transformation into metallic agglomerations during operation [179–181]. The utilization of a Cu SACs coordinated with nitrogen has demonstrated remarkable ethanol selectivity in ECO_2_RR. Operando X-ray absorption spectroscopy (XAS) has indicated that isolated Cu species underwent reversible conversion to Cu NPs, which functioned as the authentic active sites during operation [180, 181]. The stability of Cu single sites during ECO_2_RR and their ability to catalyze C–C coupling without undergoing initial structural changes that lead to Cu agglomeration is uncertain. In line with this, Zeng et al. reported a Cu coordination polymer (Cu(OH)BTA) with a stable single-site and periodic neighboring Cu centers, as illustrated in Fig. 19a [182]. The Cu(OH)BTA catalysts have been found to efficiently promote the conversion of CO_2_ to C_2+_ products, including ethylene, ethanol, acetate, and n-propanol, at a low overpotential with ethylene has been identified as the predominant product. At a potential of − 0.87 V, the total FE of C_2+_ was 73%, and a maximum FE of 57% for ethylene was achieved with a partial current density of 285 mA cm^−2^. The FE for ethanol, acetate, and *n*-propanol reached 11%, 4%, and 1%, respectively (Fig. 19b). Cu(OH)BTA demonstrated a 1.5-fold enhancement in ethylene selectivity in comparison with its metallic analogue. Operando XAS and in situ Raman as well as infrared spectroscopies (Fig. 19c–f) revealed that Cu(OH)BTA retained its structural stability and did not undergo any dynamic transformation during ECO_2_RR. Cyclic voltammetry (CV) studies were conducted in order to gain additional insight into the stability origins of the Cu(OH)BTA catalyst. The findings indicate that Cu(OH)BTA exhibits instability when subjected to Ar atmosphere during redox reactions involving Cu^2+/^Cu^1+^/Cu^0^, and such instability was observed only after 10 scans. In contrast, the use of 1 M KOH as electrolyte in the same flow cell under a CO_2_ atmosphere did not result in any redox process. The researchers postulated that the ECO_2_RR had the ability to establish a favorable local environment that acted as a protective shield for the Cu(OH)BTA molecule against the harsh alkaline environment, thereby maintaining its structural integrity. The results obtained from DFT calculations indicate that the polymer’s neighboring Cu atoms provide dual-Cu sites that are appropriately spaced. These sites facilitate the formation of an energetically suitable *OCCHO intermediate following a rate-determining step of CO hydrogenation. The energy barrier for C–C coupling was low, measuring 0.82 V, as illustrated in Fig. 19g and h.

It is noteworthy to mention that the effective conversion of CO_2_ into C_3_ products using Cu-based electrocatalysts is rarely reported. It is necessary to conduct an in-depth investigation of the reaction pathways of Cu-based electrocatalysts and their resultant products in order to gain a comprehensive understanding of the electrocatalytic CO_2_ conversion to C_3_ compounds. Chen et al. have recently reported that Cu SAs anchored in N-doped porous carbon (Cu-SA/NPC) catalysts can convert CO_2_ to ethanol, acetic acid, and acetone products at a low overpotential. Interestingly, acetone was found to be the major product, with a production rate of 336.1 μg h^−1^ and a Faradaic efficiency of 36.7%. The authors performed an investigation into the impact of copper distribution and local coordination environment of Cu SAs in order to gain insight into the ECO_2_RR to C_3_ oxygenates [183]. The DFT calculations and the experimental results revealed that the high ability of Cu-SA/NPC catalyst for producing acetone from CO_2_ reduction was ascribed to the crucial role of Cu-pyrrolic-N_4_ active sites in stabilizing the intermediates associated with acetone production and promoting C–C coupling reactions owing to the synergistic effect in Cu–N configuration.

## Summary and Outlook

This review provides an overview of the latest research advancements in Cu-based materials and their role in photocatalytic and electrocatalytic CO_2_ reduction to C_2+_ products. The primary emphasis is placed on understanding the structure–activity relationships. The economic advantages of C_2+_ products surpass those of C_1_ products, thereby underscoring the significance of developing efficient catalysts. The inherent advantages of Cu-based materials make them a promising option for CO_2_ reduction processes. However, there is a need to improve maximum selectivity and intrinsic activity for C–C bond formation, which remains a challenge in C_2+_ chemical production.

Various strategies have been employed to enhance the effectiveness and selectivity of PCO_2_RR toward C_2+_ products, including crystal phase and morphology optimization, metal doping, defect engineering, heterostructure fabrication, and bimetallic synergies. The aforementioned improvements are related to the semiconductor’s ability to effectively separate and transfer photo-induced charge carriers to the active site with minimal recombination, concentrate electrons on the active site, increase CO_2_ adsorption capacity, and facilitate C–C coupling reactions for the formation of C_2+_ products. The C–C coupling process in PCO_2_RR involves stabilizing intermediates and accumulating electrons to reduce *CO intermediates into C_2+_ products. In order to enhance the reduction capability of the photocatalyst and extend its charge lifetime, it is imperative to adequately inhibit charge recombination and precisely adjust the conduction band’s position toward a significantly negative direction.

Improving the proportion of Cu species can enhance the efficiency of Cu-based materials in PCO_2_RR to C_2+_ compounds. Nevertheless, the influence of various ratios of copper valences on its catalytic activity has not been exhaustively studied due to difficulties in precisely controlling Cu^0^, Cu^+^, and Cu^2+^ ratios in Cu-based catalysts. Therefore, modifying the structural properties of these catalysts to enhance their PCO_2_RR performance is a preferred research direction. Strategies such as pursuing controlled morphology and particle size with abundant efficient active sites as well as inducing surface defects can significantly improve the catalyst’s performance. As example, engineering the defects or oxygen/metal vacancies on the surface of copper catalyst may create more efficient active sites and allows for a continuous supply of Cu^+^ species to be available in sufficient amounts. Presently, the available methods for characterization are limited to the assessment of the microstructure and local coordination environment of the catalyst before and after the reaction. Therefore, it is necessary to employ sophisticated in situ/operando characterization methodologies to effectively to dynamically monitor the evolution of copper species on Cu-based materials under reaction conditions. Copper single-atom catalysts (Cu-SACs) exhibit great potential for effective photoconversion of CO_2_ to C_2+_ products. However, despite their promising performance, they have not been widely investigated or examined. Hence, the development of Cu-SACs that enable C–C coupling for the production of higher hydrocarbon products is necessary. Optimization of the host material, incorporation of various dopants for anchoring Cu-SAs, or modification of the photoreaction parameters may potentially facilitate the aforementioned outcome.

Despite the significant progress in PCO_2_RR to C_2+_ products, the selectivity for C_2+_ products over C_1_ products, as well as the suppression of HER, remain inadequate for practical applications. This is due to various factors such as charge recombination and inefficient reaction rates. In addition, it is a commendable goal to produce C_3+_ products which possess higher energy and value compared to C_2_ products. Despite the considerable differences in charge supply and density from electrochemistry, and the presence of a high kinetic barrier, empirical findings demonstrate the possibility of achieving CO_2_ reduction C_3+_ products through photocatalysis, providing that near theoretical efficiency is attained.

Reaction conditions can have a significant impact on product selectivity. When compared to vapor-phase CO_2_ reduction conditions, aqueous-phase CO_2_ reduction reactions offer advantages in terms of charge and mass transfer processes, which are conducive to CO_2_ reduction. However, the aqueous phase also tends to result in more pronounced competitive HER, which can lead to changes in product selectivity. Furthermore, the use of a suitable hole sacrificial agent can enhance the separation of photogenerated holes and electrons by eliminating the holes. This allows a greater number of photogenerated electrons to participate in photoreduction reactions, offering a better opportunity to produce C_2+_ products. Furthermore, to thoroughly investigate product selectivity, it is imperative to employ a robust product detection system that can both qualitatively and quantitatively measure all possible products, even those found at extremely low concentrations. Developing such a detection system is of paramount importance, as it greatly contributes to achieving more accurate studies on reaction mechanisms and product selectivity.

For ECO_2_RR to C_2+_ products, most of the electrocatalysts are made of Cu as the base due to its unique ability to facilitate the C–C coupling process. Nevertheless, attaining high FE at low overpotential for the production of C_2+_ chemicals, including ethylene and ethanol, remains a challenging task. Typically, these products are generated at higher potentials due to the necessity of surpassing the kinetic barriers of CO_2_ intermediates associated with the progression toward the C_2+_ pathway. Various research studies have been conducted to optimize the properties of Cu-based catalysts for attaining high performance in ECO_2_RR. Aspects such as morphology, surface composition, size, facet, defect density, and oxidation state are considered while designing these catalysts. The efficiency and selectivity of ECO_2_RR on Cu catalysts depend largely on various factors such as the composition of the electrocatalyst, local pH, and applied potential. The improvement of catalytic activity/selectivity in Cu-based electrocatalysts can be attributed to several factors, which results in complexity in identifying the catalytic center driving the ECO_2_RR performance. Although the supply of stable Cu^0^ and oxidized Cu (Cu^+^ or Cu^2+^) is essential during catalysis, other factors such as particle size, surface area, facet, defect, and morphological effects also play a significant role in achieving excellent ECO_2_RR performance. Thus, understanding the relationship between the Cu surface and CO_2_RR activity is necessary to design effective catalysts for ECO_2_RR in the future.

Moreover, incorporating secondary metals such as Pt, Ag, Au, or Zn into the single Cu crystal or Cu SAs catalyst has shown to be an effective strategy, as these alloys or bimetallic species have shown higher selectivity and stability toward C_2+_ products during ECO_2_RR. The role of guest metals in altering the catalyst’s electronic states, reaction kinetics, and binding energies of surface adsorbed intermediates depends on their arrangement on the Cu surface. The combination of metallic copper with secondary metals such as Au, Ag, and Zn may produce interfaces that change the electronic structure and facilitate tandem catalytic reactions, so enhancing the metals' intrinsic activity. The segregation of bimetallic phases may potentially enhance the transfer of CO from the guest metal sites responsible for CO production to the Cu sites, thereby leading to an increase in the concentration of *CO on the active Cu sites, which could lead to higher C–C coupling rates. Tailoring Cu-based electrocatalysts with support materials like carbon or MOFs not only preserves the catalyst structure but also provides beneficial synergistic effects that improve catalytic efficiency.

Furthermore, the utilization of DFT calculation is imperative in investigating the correlation between the microstructure of Cu-based catalysts and their catalytic performance, given the rapid progressions in computer technology and artificial intelligence. Additionally, the implementation of machine learning techniques can facilitate the exploration of prospective Cu-based catalysts for the purpose of CO_2_ reduction. The utilization of in situ and operando characterization techniques is crucial in recognizing the CO_2_ reduction mechanism and identifying the intermediates produced during the reaction. Regarding this matter, the use of in situ infrared and Raman spectroscopy, alongside in situ and operando XAS and APXPS, has been found to offer reliable data, thereby enabling the suggestion of more precise reaction mechanisms. The combined use of in situ and operando analysis is urged for the investigation of the complex and ongoing controversial mechanism of CO_2_ reduction reaction.

The advancement of technologies such as flow cells, GDE, and MEA has led to significant enhancements in reducing overpotential, improving Faradaic efficiency, increasing current density, and ensuring the stability of the ECO_2_RR. As a result, the integrated approach of combining catalyst design with operational technology represents a promising avenue for achieving the production of C_2+_ products at a low economic cost. It is anticipated that practical applications of ECO_2_RR will be realized in the industrial sector in the near future. Finally, this review aims to stimulate novel approaches in the design of Cu-based materials that can improve the efficiency of PCO_2_RR and ECO_2_RR in producing C_2+_ products.
